# Supplementary material for: Stem cell niche organization in the Drosophila ovary requires the ECM component Perlecan
Source: Curr Biol. 2021 Apr 26;31(8):1744–1753.e5. doi: 10.1016/j.cub.2021.01.071 (PMC8405445; doi:10.1016/j.cub.2021.01.071)
Supplement: Document S1. Figures S1–S3 [file mmc1.pdf]

Current Biology, Volume *31*

Supplemental Information

Stem cell niche organization in the *Drosophila* ovary requires the ECM component Perlecan

Alfonsa Díaz-Torres, Alicia E. Rosales-Nieves, John R. Pearson, Carmen Santa-Cruz Mateos, Miriam Marín-Menguiano, Owen J. Marshall, Andrea H. Brand, and Acaimo González-Reyes

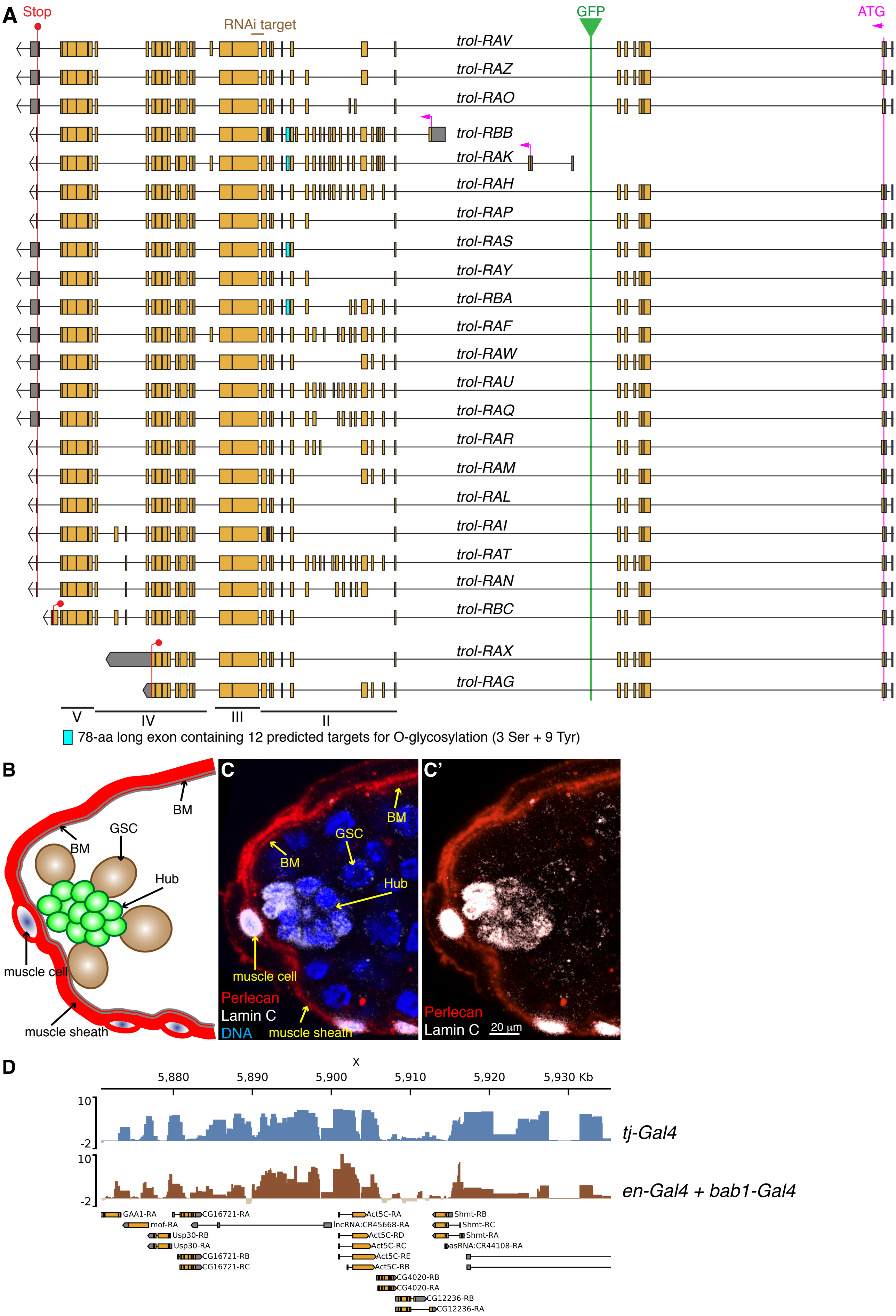
A
Stop
RNAi target
GFP
ATG
trol-RAV
trol-RAZ
trol-RAO
trol-RBB
trol-RAK
trol-RAH
trol-RAP
trol-RAS
trol-RAY
trol-RBA
trol-RAF
trol-RAW
trol-RAU
trol-RAQ
trol-RAR
trol-RAM
trol-RAL
trol-RAI
trol-RAT
trol-RAN
trol-RBC
trol-RAX
trol-RAG
V
IV
III
II
78-aa long exon containing 12 predicted targets for O-glycosylation (3 Ser + 9 Tyr)
B
BM
GSC
BM
Hub
muscle cell
muscle sheath
C
BM
GSC
BM
Hub
muscle cell
Perlecan
Lamin C
DNA
muscle sheath
C'
Perlecan
Lamin C
20 µm
D
X
5,880
5,890
5,900
5,910
5,920
5,930 Kb
10
-2
tj-Gal4
10
-2
en-Gal4 + bab1-Gal4
GAA1-RA
CG16721-RA
mof-RA
Usp30-RB
Usp30-RA
CG16721-RB
CG16721-RC
Act5C-RA
lncRNA:CR45668-RA
Act5C-RD
Act5C-RC
Act5C-RE
Act5C-RB
CG4020-RB
CG4020-RA
CG12236-RB
CG12236-RA
Shmt-RB
Shmt-RC
Shmt-RA
asRNA:CR44108-RA

Figure S1: Organisation of the *trol* gene. Perlecan accumulation in the male GSC niche. Transcription of the *Actin 5c (Act5c)* gene in different somatic cell types of the adult ovary. Related to Figures 1 and 2. (A) According to JBrowse (FlyBase2020_04), the *trol* gene can give rise to 23 different predicted isoforms. The ATGs corresponding to the long, intermediate and short versions, the STOP codons, the target sequence for the *trol RNAi* construct and the insertion point of the GFP exon are shown for all isoforms. Only the large isoforms are expected to incorporate GFP. The Perlecan antibody used in this study was raised against domain V of the protein. *Drosophila trol* lacks Domain I of the human orthologue gene. Exon in light blue is specific for only four isoforms and it is predicted to be O-glycosylated in 12 out of 78 residues. The RNAi target is shown in light brown. All isoforms should be targets of the interference construct. **(B)** Schematic representation of the *Drosophila* testis GSC niche. The anterior tip is home to the hub and a number of male GSCs attached to the hub cells. Each GSC is flanked by two somatic stem cells (cyst stem cells; not shown). The niche is surrounded by a basement membrane (BM) and an external muscle sheath. **(C)** Confocal image showing the tip of a *Drosophila* testis. Hub cells accumulate Lamin C (white) in a similar manner to CpCs. However, Perlecan (red) is not detected in the hub, even though it accumulates in the BM and the muscle sheath. Muscle cells also express nuclear Lamin C. DNA is shown in blue (Hoechst staining). **(D)** Cell-type-specific profiling of *Act5c* expression in TF cells, CpCs and ECs (*en-Gal4 + bab1-Gal4*) or in most of the somatic cells of the adult ovary (*tj-Gal4*) using the TaDa technique. Scale bars represent log2 ratio change between Dam-Pol II and Dam (reference) samples. The data are scaled so that the Pol II occupancy between the two different groups of cell types should be equivalent. Scale bar= 20µm.

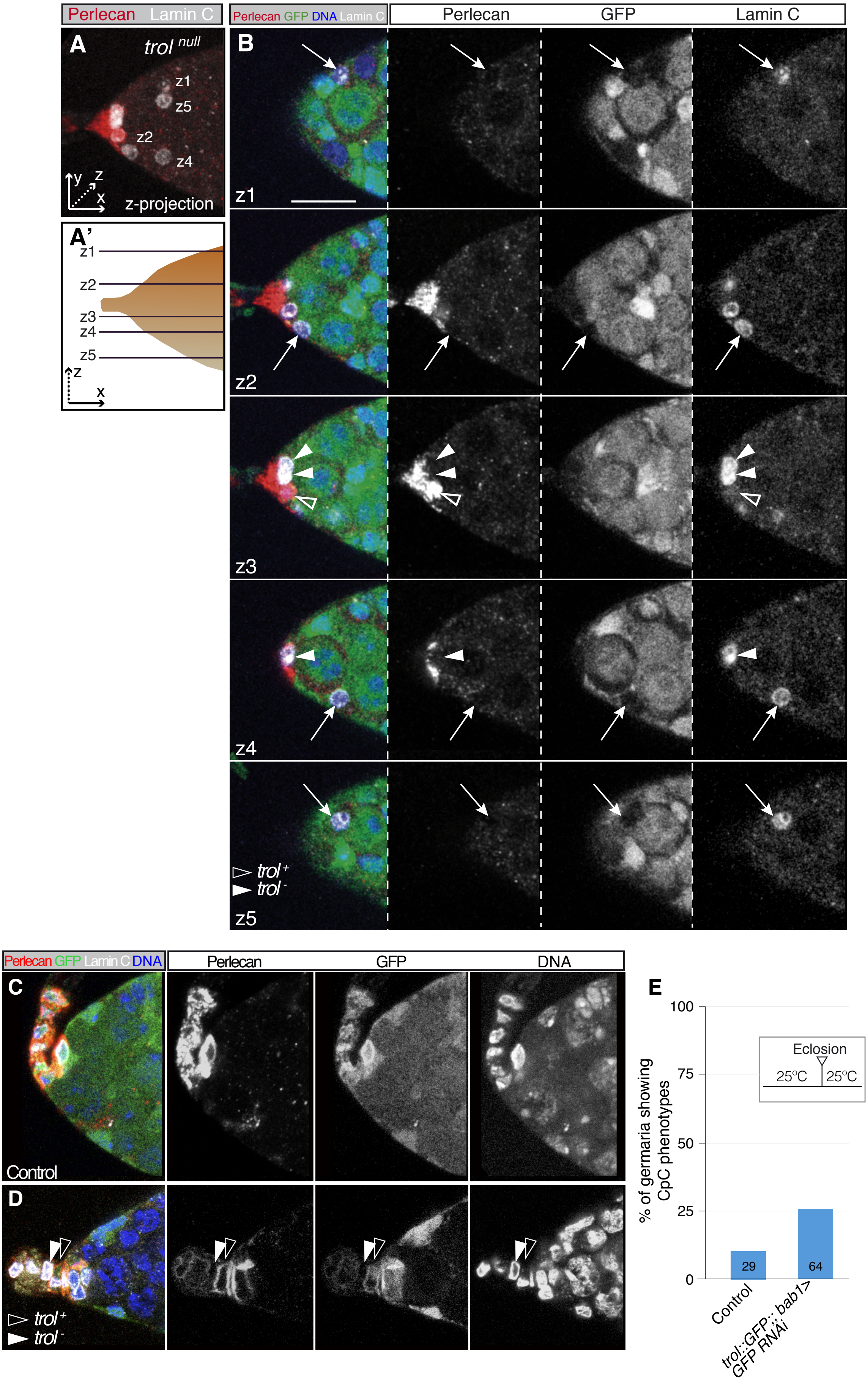

Perlecan Lamin C
A
trol null
z1
z5
z2
z4
z-projection
A'
z1
z2
z3
z4
z5
Perlecan GFP DNA Lamin C
Perlecan
GFP
Lamin C
B
z1
z2
z3
z4
z5
trol+
trol-
Perlecan GFP Lamin C DNA
Perlecan
GFP
DNA
C
Control
D
trol+
trol-
E
% of germaria showing CpC phenotypes
100
75
50
25
0
Eclosion
25°C
25°C
29
64
Control
trol::GFP;; bab1> GFP RNAi

Figure S2: Loss of *trol* activity induces cap cell displacement. Related to Figure 3. (A) Z-projection (top view) of a mosaic germarium containing several *trol* mutant CpCs. At least four of the *trol*$^{-}$ cells are not associated with the CpC rosette found at the base of the terminal filament. **(A')** Representation showing the different z-planes used in this analysis (lateral view). **(B)** Individual optical sections displaying the spatial arrangement of the mutant cells with respect to the anterior CpC rosette. CpCs are labelled with Lamin C. *trol* mutant cells are marked by the lack of GFP and Perlecan staining. Plane z3 shows a *trol*$^{+}$ CpC with high Perlecan levels. Its Lamin-C staining can be observed better in plane z2. **(C)** Z-sections of a control and **(D)** a mosaic germarium containing a *trol*$^{-}$ TF cell stained to visualise Perlecan, GFP, Lamin C and DNA. The mutant TF cell (GFP^{-}; solid arrowhead) shows a noticeable reduction in Perlecan levels. **(E)** Quantification of the number of abnormal niches containing displaced CpCs in control and in experimental (*trol::GFP;; bab1>GFP RNAi*) germaria grown at 25°C. Numbers in bars refer to number of germaria analysed. Arrows: displaced *trol*$^{-}$ CpCs; empty arrowheads: control CpCs in the rosette; solid arrowheads: mutant CpCs in the rosette. (A, B) Clones were induced using the *bab1-Gal4/UASt-flp* system. (C) To increase the occurrence of mitotic recombination, in addition to inducing *flp* expression with the *bab1-Gal4/UASt-flp* system, flies of the appropriate genotype were subjected to heat-shock to express *flp* from the *hs-flp* construct (see STAR METHODS in the main text). Scale bar = 20µm.

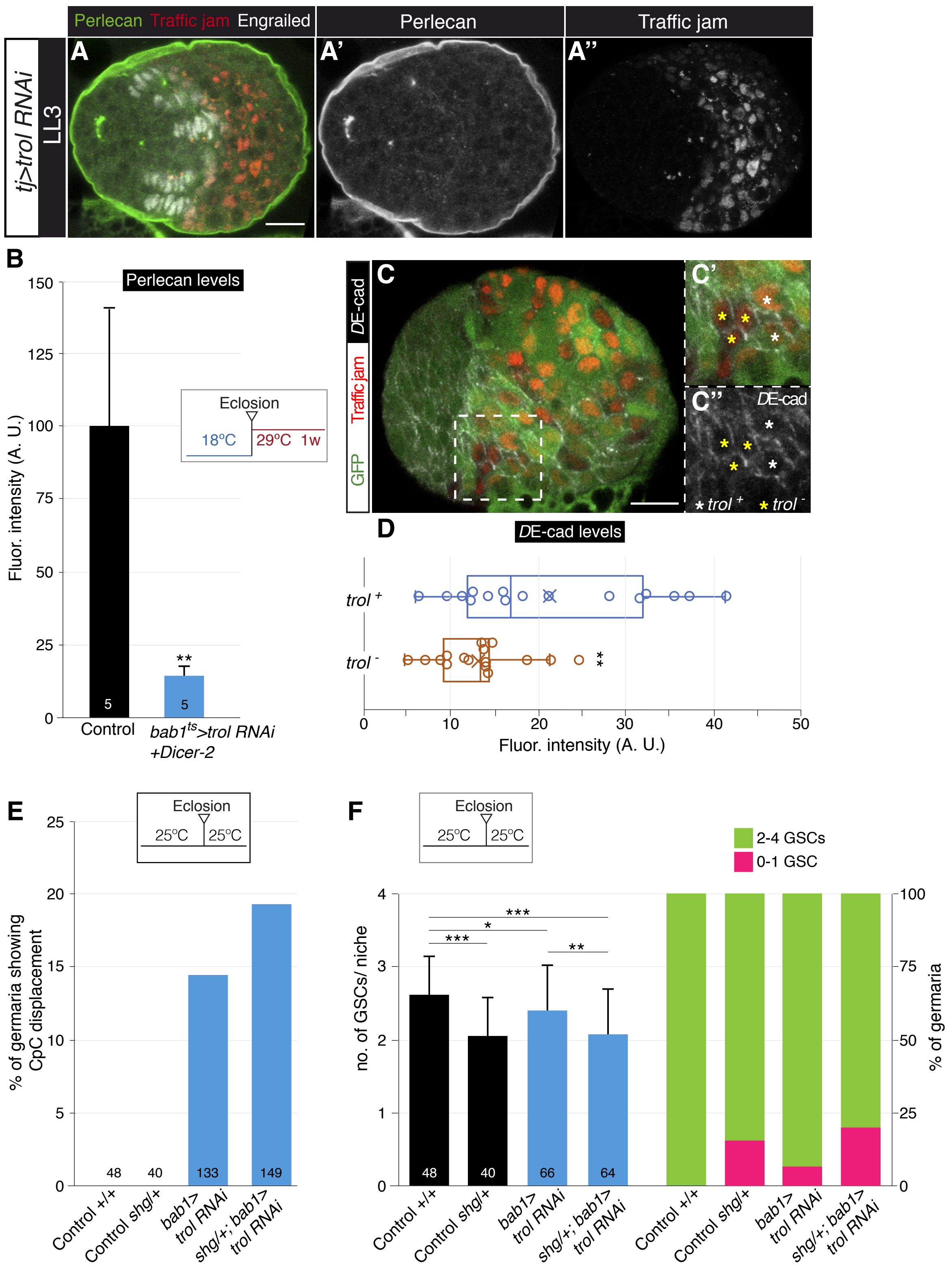

Perlecan Traffic jam Engrailed
Perlecan
Traffic jam
A
A'
A''
tj>trol RNAi
LL3
B
Perlecan levels
Fluor. intensity (A. U.)
Eclosion
18ºC
29ºC 1w
Control
bab1ts>trol RNAi +Dicer-2
C
C'
C''
DE-cad
Traffic jam
GFP
trol +
trol -
D
DE-cad levels
Fluor. intensity (A. U.)
E
Eclosion
25ºC
25ºC
% of germaria showing CpC displacement
Control +/+
Control shg/+
bab1> trol RNAi
shg/+; bab1> trol RNAi
F
no. of GSCs/ niche
% of germaria
2-4 GSCs
0-1 GSC

Figure S3: Low Perlecan levels in *tj>trol RNAi* gonads. Quantification of Perlecan levels in *bab1^{ts}>trol RNAi+dicer* niches. Quantification of *D*E-cadherin levels in mosaic gonads. Genetic interaction between *shotgun* and *trol* in adult niches. Related to Figure 4. (A) Z-projection of a *tj>trol RNAi* LL3 gonad stained to visualise Perlecan, Traffic jam and Engrailed (to label TF cells). As an indication of the effectiveness of the *trol* RNAi tool, notice the strong reduction in Perlecan levels at the base of the TFs and in the PGC/ISC area. This experimental gonad corresponds to the control shown in Figure 4C. **(B)** Quantification of anti-Perlecan immunofluorescence signal in control and *bab1^{ts}>trol RNAi + Dicer-2* germaria grown at 18°C and kept for 1 week at 29°C upon eclosion. **(C)** Z-projection of a mosaic LL3 gonad stained to visualise Traffic jam, GFP and *D*E-cadherin. In this particular example, three mutant Tj-positive cells at the anterior limit of the germline cluster (considered to be prospective CpCs; yellow asterisks) are compared to neighbour control cells (white asterisks). **(D)** Quantification of *D*E-cadherin levels at *trol^{+}/trol^{+}* or *trol^{-}/trol^{-}* cell boundaries was performed on CpC or TF cells of LL3 gonads. *trol^{-}* cells localise significantly lower *D*E-cadherin amounts at their surfaces than *trol^{+}* cells. To allow for paired comparisons, measurements were taken from neighbouring control and experimental cells. P values of two-tailed, paired t-tests considered statistically significant between control and experimental samples are indicated (**: P≤0.005). The mean (cross) and median (line across box) for each of the samples are shown. We quantified 16 *trol^{+}* and 16 *trol^{-}* CpC or TF cell boundaries from 9 gonads. Clones were induced using the *hs-flp/FRT* system. **(E)** Percentage of control and experimental germaria showing displaced CpCs. **(F)** Quantification of the number of GSCs per niche and distribution of germaria containing 0-1 or 2-4 GSCs in control and experimental germaria. P values of two-tailed, unpaired t-tests considered statistically significant between control and experimental samples are indicated (*: P≤0.05, **: P≤0.005, ***: P≤0.0005). Numbers in bars refer to number of germaria analysed. Scale bars = 10µm.
