# Supplementary material for: Stem cell niche organization in the Drosophila ovary requires the ECM component Perlecan
Source: Curr Biol. 2021 Apr 26;31(8):1744–1753.e5. doi: 10.1016/j.cub.2021.01.071 (PMC8405445; doi:10.1016/j.cub.2021.01.071)
Supplement: Document S2. Article plus supplemental information [file mmc7.pdf]

Current Biology

Report

Stem cell niche organization in the *Drosophila* ovary requires the ECM component Perlecan

Highlights

- The *Drosophila* ovarian niche contains a Perlecan-rich interstitial matrix
- Niche cells express and secrete specific Perlecan isoforms
- Absence of *trol* results in aberrant niches containing fewer niche and stem cells
- *trol* regulates *D*E-cadherin levels in larval and adult niche cells

Authors

Alfonsa Díaz-Torres,
Alicia E. Rosales-Nieves,
John R. Pearson, ..., Owen J. Marshall,
Andrea H. Brand,
Acaimo González-Reyes

Correspondence

agonrey@upo.es

In Brief

Tissues host stem cells in niches that normally contain extracellular matrix (ECM). Díaz-Torres et al. identify the ECM molecule Perlecan as an essential component of the ovarian niche in *Drosophila*. Results reveal the importance of Perlecan for proper niche morphogenesis during pre-adult development and for tissue homeostasis in the adult female.

Díaz-Torres et al., 2021, Current Biology *31*, 1744–1753
April 26, 2021 © 2021 The Authors. Published by Elsevier Inc.
https://doi.org/10.1016/j.cub.2021.01.071

CellPress

CellPress
OPEN ACCESS

Current Biology

Report

Stem cell niche organization in the *Drosophila* ovary requires the ECM component Perlecan

Alfonsa Díaz-Torres,[1,5] Alicia E. Rosales-Nieves,[1,6] John R. Pearson,[1,7] Carmen Santa-Cruz Mateos,[1] Miriam Marín-Menguiano,[1] Owen J. Marshall,[2,3] Andrea H. Brand,[4] and Acaimo González-Reyes[1,8,*]

[1]Centro Andaluz de Biología del Desarrollo, CSIC/Universidad Pablo de Olavide/JA, Carretera de Utrera km 1, 41013 Sevilla, Spain
[2]The Gurdon Institute and Department of Physiology, Development and Neuroscience, University of Cambridge, Downing Street, Cambridge CB2 1QN, UK
[3]Menzies Institute for Medical Research, University of Tasmania, 17 Liverpool St, Hobart, TAS 7000, Australia
[4]The Gurdon Institute and Department of Physiology, Development and Neuroscience, University of Cambridge, Downing Street, Cambridge CB2 1QN, UK
[5]Present address: Microscopy Unit, Centro Nacional de Investigaciones Cardiovasculares (CNIC), 28029 Madrid, Spain
[6]Present address: Instituto de Biomedicina de Sevilla (IBiS), Hospital Universitario Virgen del Rocío/CSIC/Universidad de Sevilla, 41011 Seville, Spain
[7]Present address: Centro Andaluz de Nanomedicina y Biotecnología (BIONAND), C/ Severo Ochoa 35, Parque Tecnológico de Andalucía, 29590 Campanillas (Málaga), Spain
[8]Lead contact
*Correspondence: agonrey@upo.es
https://doi.org/10.1016/j.cub.2021.01.071

SUMMARY

Stem cells reside in specialized microenvironments or niches that balance stem cell proliferation and differentiation.[1,2] The extracellular matrix (ECM) is an essential component of most niches, because it controls niche homeostasis, provides physical support, and conveys extracellular signals.[3–11] Basement membranes (BMs) are thin ECM sheets that are constituted mainly by Laminins, Perlecan, Collagen IV, and Entactin/Nidogen and surround epithelia and other tissues.[12] Perlecans are secreted proteoglycans that interact with ECM proteins, ligands, receptors, and growth factors such as FGF, PDGF, VEGF, Hedgehog, and Wingless.[13–18] Thus, Perlecans have structural and signaling functions through the binding, storage, or sequestering of specific ligands. We have used the *Drosophila* ovary to assess the importance of Perlecan in the functioning of a stem cell niche. Ovarioles in the adult ovary are enveloped by an ECM sheath and possess a tapered structure at their anterior apex termed the germarium. The anterior tip of the germarium hosts the germline niche, where two to four germline stem cells (GSCs) reside together with a few somatic cells: terminal filament cells (TFCs), cap cells (CpCs), and escort cells (ECs).[19] We report that niche architecture in the developing gonad requires *trol,* that niche cells secrete an isoform-specific Perlecan-rich interstitial matrix, and that *D*E-cadherin-dependent stem cell-niche adhesion necessitates *trol*. Hence, we provide evidence to support a structural role for Perlecan in germline niche establishment during larval stages and in the maintenance of a normal pool of stem cells in the adult niche.

RESULTS AND DISCUSSION

CpCs organize into a 6–8 cell rosette positioned at the base of the TF. Both cell types are connected by the "transition cell."[20] TFCs and CpCs can be distinguished from their characteristic shape, Engrailed (En) expression, and high Lamin C contents. GSCs are anchored to the adjacent CpC rosette by adherens junctions, and this adhesion prevents GSC loss from the niche[21] (Figures 1A and 1A′).

The *Drosophila* ovarian niche possesses a specialized extracellular matrix

We determined the pattern of expression of Perlecan in relation to Collagen IV, Laminin β, and Nidogen in the ovarian niche and early egg chambers (Figures 1B–1F). As previously reported,[11] Collagen IV::GFP (ColIV::GFP)[22,23] is strongly expressed in the matrix surrounding the niche, and a discrete signal is detected in the interstitial space between TFCs and CpCs (Figure 1B). Laminin β and Nidogen display similar patterns of expression except that their interstitial signal is even less conspicuous than that of ColIV::GFP (Figures 1C and 1D). They all are expressed in the BM of young egg chambers.

The *terribly reduced optic lobes* (*trol*) gene, which encodes the Perlecan proteoglycans in *Drosophila*,[17,18,24,25] is predicted to produce 23 different isoforms transcribed from three different promoters, giving rise to one short isoform (RBB), one intermediate isoform (RAK), and 21 long isoforms, two of which are truncated at their 3′ ends (*trol-RAG* and *trol-RAX*). The rest of the

 © 2021 The Authors. Published by Elsevier Inc.
This is an open access article under the CC BY license (http://creativecommons.org/licenses/by/4.0/).

CellPress
OPEN ACCESS

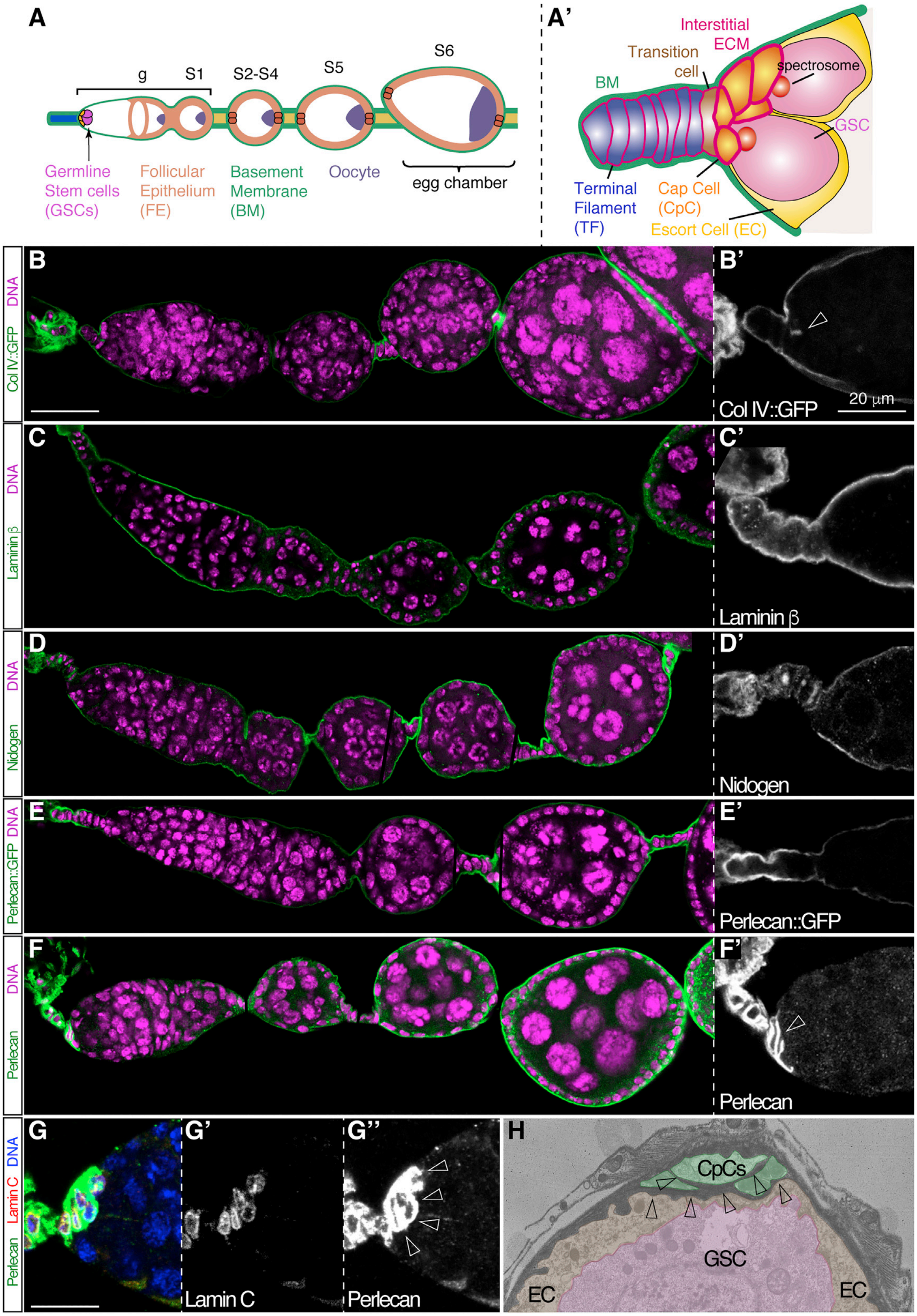

Figure 1. Distribution of BM components in control ovarioles

(A and A′) Scheme of a control ovariole showing the germarium and the GSCs within, the cellular organization of the follicular epithelium, the posteriorly placed oocyte, and the surrounding BM. Egg chambers of different stages (S) are shown. The magnification in (A′) depicts GSC niche components.

(legend continued on next page)

isoforms contain all of the conserved domains found in *Drosophila* Perlecan (domains II to V of the human homologue; Figure S1A) except *trol-RAG* and *trol-RAX*. To define Perlecan distribution in the niche and in early oogenesis, we used a *trol::GFP* line where only long isoforms are targeted. Similarly to Laminin, ColIV::GFP, and Nidogen, Perlecan::GFP accumulates in the BM surrounding the ovariole without detectable expression between niche cells (Figure 1E). A Perlecan antibody that recognizes domain V of the protein localized to the BM around the niche and from S3 onwards, whereas in S1 and S2 egg chambers it strongly accumulates in vesicle-like dots inside the follicle cells.[26] In clear contrast to the distribution of Perlecan::GFP, Perlecan antibody localized strongly around TFCs, in CpC-CpC boundaries, and in CpC-GSC contacts, albeit less pronouncedly in the latter. This suggests the accumulation of a specialized interstitial matrix around TFCs and CpCs, an idea further confirmed by the presence of deposits of electron-dense material in the intercellular spaces between CpCs and the CpC-GSC boundaries in transmission electron micrographs (Figures 1F–1H). We conclude that the short and/or the intermediate Perlecan isoforms accumulate specifically in the interstitial matrix of the GSC niche, whereas the long isoforms are incorporated mainly into the BM.

The male GSC niche exhibits a number of similarities with its female equivalent, including a cluster of highly packed hub cells that resemble CpCs as they act as a signaling center.[27] Male GSCs surround the hub cells and are flanked by somatic cyst stem cells. As in the case of the ovariole, the whole structure is surrounded by BM and a muscle sheath (reviewed in Greenspan et al.[28]). However, in spite of these similarities, hub cells were not surrounded by a deposition of Perlecan protein, even though Perlecan accumulated in the basement membrane and the muscle sheath of the apical end of the testis (Figures S1B and S1C).

Gene expression profiling identifies niche-specific *trol* isoforms

Next, we used Targeted DamID (TaDa) to test whether the presumptive niche-specific localization of Perlecan variants corresponded with differential expression of *trol* isoforms. TaDa utilizes the Gal4/UAS system to express Dam-Pol II, a fusion of the Dam methylase and the RNA polymerase II core subunit RpII215, to define cell-type-specific transcriptomes.[29] In combination with *tub-Gal80*^{ts}, we first expressed Dam-Pol II in adult niche cells by using *en-Gal4* and *bab1-Gal4*. As a positive control for *trol* isoform transcription, we expressed Dam-Pol II in most of the somatic cells of the ovary with *tj-Gal4* (Figure 2A).[20,30] To control for non-specific methylation, we expressed the Dam methylase alone with the same drivers. Due to the reduced numbers of target cells for the *en-* and *bab1-Gal4* drivers in comparison with *tj-Gal4* drivers, we combined their profiles to define those genes expressed in niche cells. Only genes with an FDR (false discovery rate of enriched Pol II occupancy) of <0.01 were considered. As a positive control, we first checked that the expression of the ubiquitously expressed gene *Act5c* was detected in all datasets (Figure S1D).

We identified 5,032 genes, 1,614 specific to the *tj*$^{+}$ cells and 877 exclusive to the *en+bab1* sets that could represent niche-specific genes expressed in TFCs and likely enriched in CpCs (Figure 2A and Data S1). Surprisingly, niche genes such as *dpp*, *gbb*, *upd*, and *hh*[19,31–35] were not among the identified genes. It is conceivable that during the initial procedure of DNA extraction, DNA shearing could not be completely avoided due to the high cell numbers in the ovary, resulting in higher levels of non-specific signal. Hence, low-level expressed genes could be under-represented and could fall below the implemented FDR <0.01 threshold. Nevertheless, we identified several niche genes including *hopscotch*, *shotgun*, and several of the *innexin* genes,[21,36,37] indicating that our approach, albeit with some limitations, is a valid strategy for defining the niche transcriptome.

We used Pol II occupancy profile peaks and direct comparison with the predicted transcription start (TSS) and transcription end (TES) sites of the different isoforms to define the *trol* variants transcribed in niche cells. *tj-Gal4*-expressing cells utilize all three TSSs and both TESs, indicating that these cells actively transcribed all four isoform types (Figures 2B and 2C; Figure S1A). In *en-Gal4* + *bab1-Gal4* cells, which did not seem to express the intermediate RAK isoform nor the RAG and RAX truncated long isoforms, the promoters giving rise to long and the short (RBB) isoforms were active. Because TFCs express Perlecan::GFP and CpCs accumulate Perlecan but not Perlecan::GFP, our results indicate that CpCs mainly express *trol-RBB*, the short isoform. Although we cannot exclude that post-transcriptional modifications of the Perlecan protein affect its stability and/or localization and, hence, that the accumulation of Perlecan or Perlecan:GFP could occur away from the producing cells, we find this unlikely, because CpCs lacking the *trol* gene do not accumulate Perlecan (see below), which suggests a very limited diffusion of the protein from the producing CpCs.

trol activity is required for niche organization

To test whether Perlecan had a role in niche architecture, we used RNA interference to decrease *trol* function. TFs and CpCs from *bab1-Gal4*, *UAS-trol RNAi* (*bab1>trol RNAi*) females grown at 25°C showed a ~7-fold reduction in Perlecan proteins when

(B and B′) Collagen IV distribution, as shown by the localization of the fusion protein Col IV::GFP, encoded by the *viking::GFP* (*vkg::GFP*) gene.
(C and C′) Laminin ß expression pattern as shown with an anti-Laminin ß antibody.
(D and D′) Nidogen expression, detected with an anti-Nidogen antibody.
(E and E′) Localization of the Perlecan::GFP fusion protein, encoded by *trol::GFP*.
(F and F′) Perlecan accumulation, as visualized with an anti-Perlecan antibody. Note that the Perlecan::GFP protein trap clearly accumulates in the BM, whereas the anti-Perlecan antibody shows strong staining in TFCs and CpCs and in the BM of S3 and later egg chambers. The Perlecan staining is so much stronger in the interstitial matrix between niche cells that the confocal gain has to be reduced to avoid saturating the image. Thus, the weaker anti-Perlecan signal in the niche BM often goes undetected.
(G–G′′) Perlecan accumulates in the TF and CpC rosettes, which are labelled with Lamin C.
(H) Transmission electron micrograph of a control niche. CpCs, ECs, and GSCs are pseudo-colored. Note the electron-dense material that surrounds the entire germarium tip and is also found enclosing the CpCs. Empty arrowheads point to the interstitial matrix present in the niche.
(B)–(F) correspond to different z-sections stitched together in a single plane; scale bar in (B)–(F), 50 μm. Scale bar in (G), 15 μm. See also Figure S1.

CellPress
OPEN ACCESS

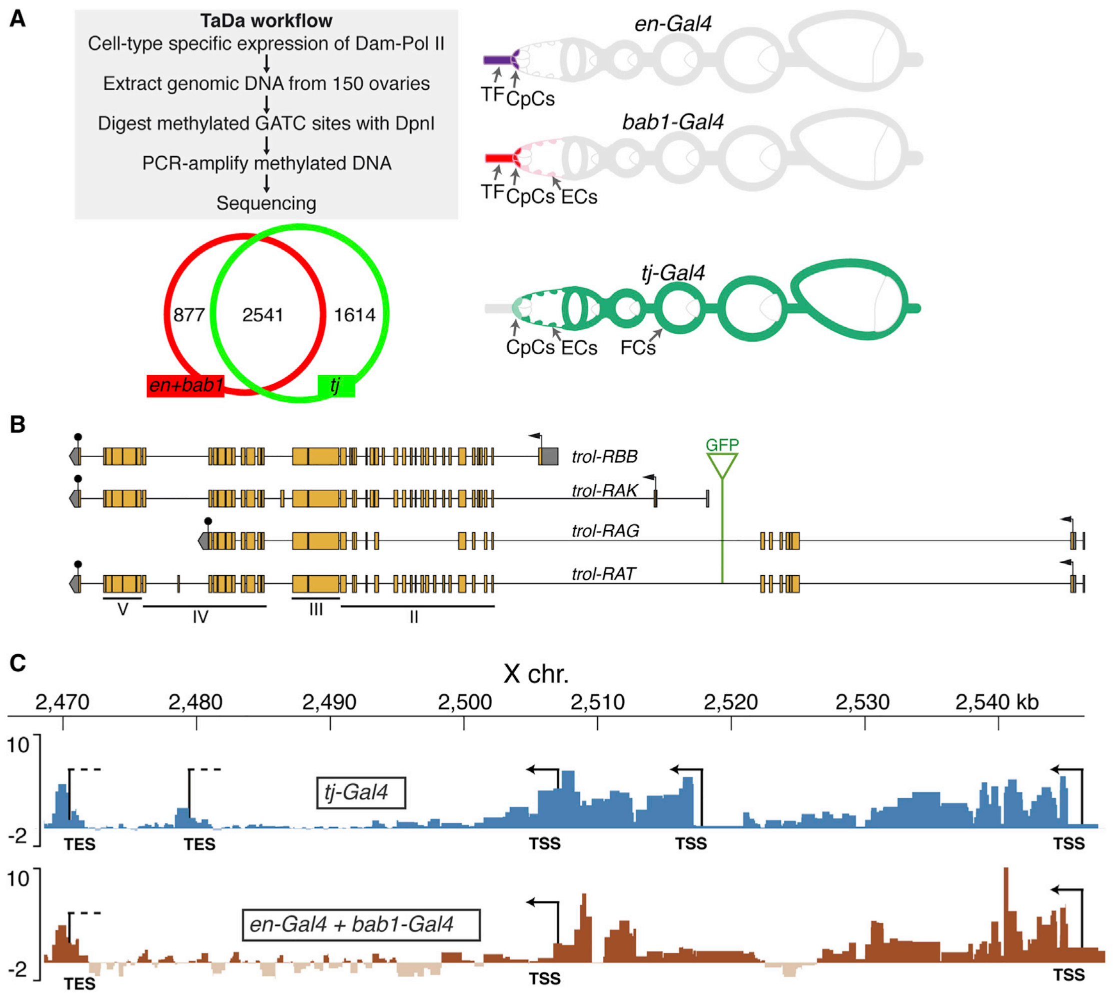

Figure 2. Transcription of *trol* isoforms in different somatic cell types of the adult ovary

(A) Workflow for the TaDa analysis. The Dam-Pol II fusion protein was expressed in TFCs, CpCs, and ECs with the *engrailed-Gal4* and *bric-a-brac 1-Gal4* (*en-Gal4* + *bab1-Gal4*) lines. The *traffic jam-Gal4* (*tj-Gal4*) line is expressed in most of the somatic cells of the ovary except the TF.

(B) The 23 isoforms encoded by the *trol* gene can be grouped into four types: one short isoform (*trol-RBB*), an intermediate isoform (*trol-RAK*), two long isoforms with truncated 3′ ends (*trol-RAG* and *trol-RAX*) and 19 long versions. Of the latter groups, only *trol-RAG* and the long isoform *trol-RAT* are shown. Compared to its human orthologue, *Drosophila* Perlecan proteins contain domains II–V but lack domain I. The insertion point of the artificial GFP-encoding exon, the translation initiation codons (arrows), and the translational stop codons (solid black circles) are shown.

(C) Differential Pol II occupancy of *trol* in tj^+ cells (*tj-Gal4*) or in niche cells (*en-Gal4* + *bab1-Gal4*) of the adult ovary. Scale bars represent log2 ratio change between Dam-Pol II and Dam (reference) samples. The data are scaled so that the Pol II occupancy between the two different cell types should be equivalent. *tj-Gal4* cells express all four types of isoforms and use the three TSSs and both TESs. *en-Gal4* + *bab1-Gal4* cells transcribe neither the intermediate *trol-RAK* nor the long, truncated versions *trol-RAG* and *trol-RAX.* (See Figure S1A for a representation of all *trol* isoforms). The peaks are larger at the beginning of transcription due to the longer duration of the transcription initiation phase, which increases the chance of Pol II binding to the TSS regions and thus the possibility of being identified in the TaDa analysis. See also Data S1 and Figure S1.

compared to controls (Figures 3A and 3B). These *bab1>trol RNAi* germaria displayed a number of mutant phenotypes. First, the number of CpCs in experimental rosettes was lower than in controls (7.1 ± 1.21 CpCs in controls; 6 ± 1.54 in *bab1>trol-RNAi*; Figure 3C). Second, in 22% of experimental germaria we observed abnormal CpC rosettes in which individual, or groups of, Lamin C^+ cells were displaced from the base of the TF. These displaced CpCs also expressed Engrailed, another CpC marker (Figures 3A and 3D; Video S1). This phenotype was found even in germaria from freshly eclosed females. Third, the number of GSCs/niche was also significantly reduced in *bab1>trol RNAi* flies (2.70 ± 0.56 in controls; 2.37 ± 0.71 in experimental ones; Figure 3E). Furthermore, 10% of *bab1>trol RNAi* germaria that were analyzed contained 0 or 1 GSC/niche, whereas all of the control niches hosted ≥2 GSCs (Figure 3E). These results confirm that *trol* is required to maintain GSC niche integrity and to preserve a normal pool of stem cells within it.

Next, we generated *trol* null CpCs during larval and pupal stages, which is when CpC precursors proliferate and mitotic clones can be induced. We observed that $trol^-$ CpCs (recognized by the round shape of their nuclei, the loss of GFP signal, and their expression of Lamin C) could be found displaced from

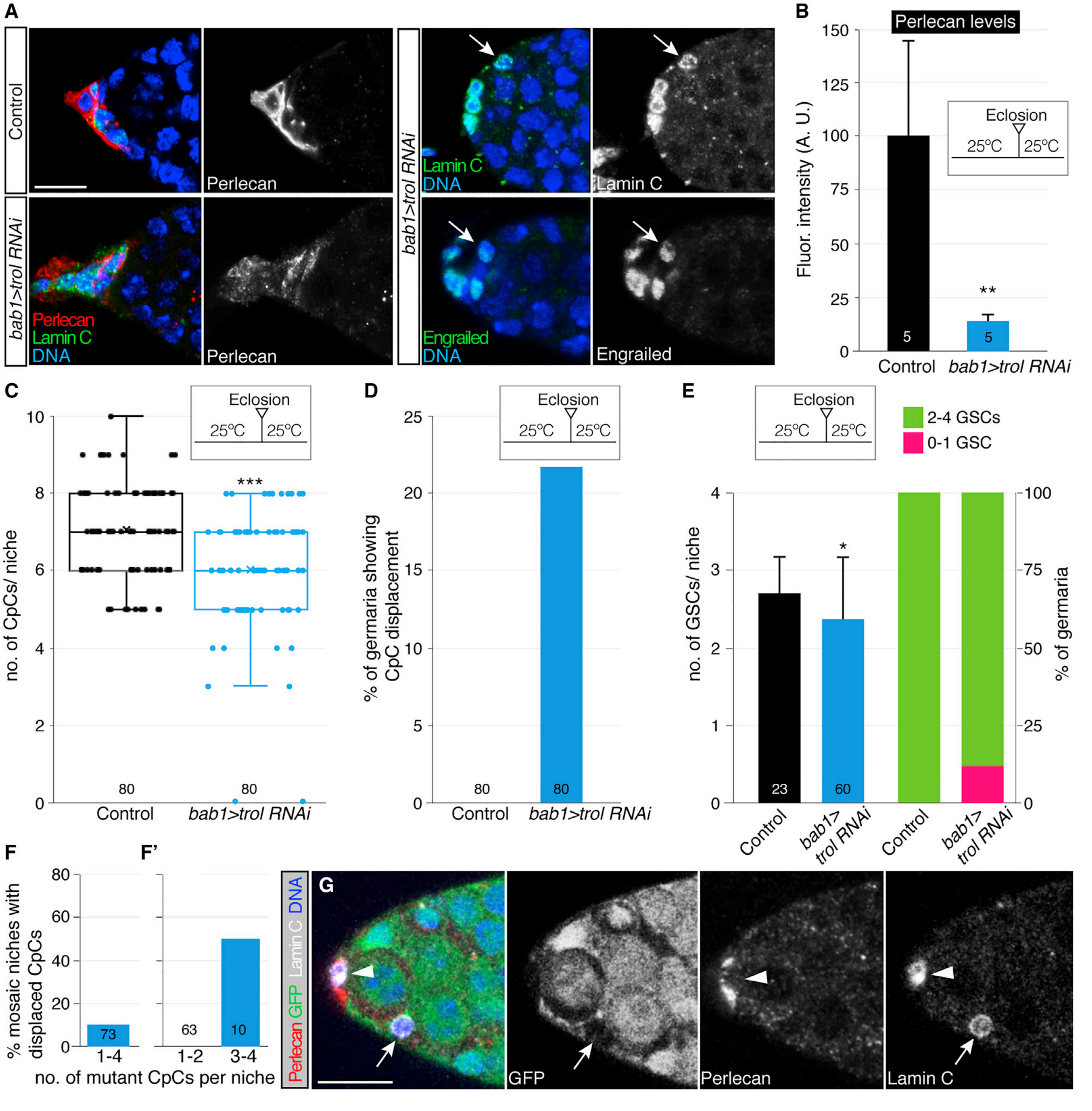

Figure 3. Loss of *trol* activity induces CpC displacement

(A) Immunodetection of Perlecan, Lamin C, and Engrailed in control and *bab1>trol RNAi* germaria. *bab1>trol RNAi* germaria show reduced Perlecan levels and displaced CpCs (arrows). Images are maximum projections of two sections along the Z-axis.

(B) Quantification of the Perlecan immunofluorescence signal in control and *bab1>trol RNAi* germaria.

(C) Graph displaying the number of CpCs per rosette in niches of the above genotypes.

(D) Percentage of control and experimental germaria showing displaced CpCs.

(E) Quantification of the number of GSCs per niche and distribution of germaria containing 0–1 or 2–4 GSCs in control and experimental germaria.

(F) Graph representing the analysis of 73 mosaic germaria containing 1 to 4 *trol* mutant CpCs. 6.7% of mosaic niches show displaced CpCs.

(F′) Niches with 1–2 mutant CpCs do not show displaced cells, whereas ~50% of those containing 3–4 $trol^-$ CpCs display the phenotype. The appearance of displaced CpCs thus requires at least 3 $trol^-$ CpCs in a given niche.

(G) Single z-section of a mosaic germarium containing a displaced *trol* mutant CpC (see Figure S2 and Video S2 for further details).

(A–E) Germaria from flies grown at 25°C. Arrows, displaced $trol^-$ CpCs; arrowhead, mutant CpC in the rosette. Clones were induced by using the *bab1-Gal4/UASt-flp* system. p values of two-tailed, unpaired t tests considered statistically significant between control and experimental samples are indicated (*$p \leq 0.05$; **$p \leq 0.005$; ***$p \leq 0.0005$). Numbers in bars refer to number of germaria analyzed. Scale bars, 10 μm. See also Figure S2 and Videos S1 and S2.

their normal location at the base of the TF in 7% of mosaic germaria (Figures 3F and 3G; Figure S2A; Video S2). A detailed analysis of the aberrant niches confirmed that only those germaria containing $\geq$3 mutant CpCs showed the CpC displacement phenotype (50% of niches with $\geq$3 mutant CpCs; Figure 3F′). Mutant cells—even those still located in the rosette—failed to accumulate Perlecan, indicating that CpCs autonomously produced the surrounding Perlecan (Figure S2B, planes z3 and z4). Thus, our results strongly suggest that Perlecan protein is required for the proper establishment and/or maintenance of the CpC rosette and, as a consequence, for hosting a normal GSC pool within the niche. The fact that at least three *trol*$^-$ CpCs are needed to observe the displacement phenotype suggests that Perlecan secreted from neighboring *trol*$^+$ CpCs can rescue the loss of *trol* activity in individual CpCs. TFCs mutant for *trol* also display reduced Perlecan levels, indicating that, at least partially, TFCs secrete Perlecan (Figures S2C and S2D). Finally, we generated *trol::GFP;; bab1-Gal4/UAS-sh:GFP* (*trol::GFP;; bab1>GFP RNAi*) flies and grew them at 25°C to deplete the developing gonads and the adult niches of long Perlecan isoforms. One-week-old control germaria showed alterations in the organization of niche cells in 13.8% of the samples, whereas experimental germaria displayed aberrant niches containing displaced CpCs, individual or in clusters, in 25.6% of the cases (Figure S2E).

trol is required for niche establishment during larval stages

We then studied whether *trol* function was required during niche formation in third instar larvae/early pupae. Larval gonads can be divided into three regions: an anterior one where TFCs and CpC precursors are located; a central region, which houses the primordial germ cells (PGCs) intermingled with their somatic cell neighbors (ISCs); and a posterior region.[38] ISCs and the future CpCs express the Traffic-jam protein[39] (Figure 4). At late third-instar larval stage, TFCs differentiate and arrange into the separate stalks that will constitute the ovarioles' anterior tip. TFs appear in a morphogenetic wave from medial to lateral positions across the gonad until early pupal development.[39,40] At the larval-to-pupal transition, some of the somatic cells juxtaposed with the TFs differentiate into CpCs or ECs, PGCs located next to the newly formed CpCs convert into GSCs, and the niche becomes a functional unit that hosts on average 2–4 GSCs (Figure 4A).

Perlecan localization in developing gonads is consistent with a role during niche formation. At mid- and late-third instar larval gonads (ML3 and LL3) Perlecan is found lining the gonad periphery, at the interface with the fat body, but it is also detectable internally, within the gonadal cells. We observed regions of strong localization between the anterior somatic cells and in the area where the PGCs gather together. Importantly for our studies, Perlecan accumulation was detected between the developing niche and the PGCs, delimiting both regions in ML3 (Figure 4B). We found that this interstitial accumulation of Perlecan increased over time and was abundantly distributed in the PGC region and in patches in between somatic cells at LL3. Perlecan::GFP showed a similar distribution (Figure 4C; Video S3).

To assess the importance of Perlecan for niche establishment in larvae, we removed Perlecan from large regions of the gonad with RNA interference. First, we used *traffic jam-Gal4*, *UAS-trol RNAi* (*tj>trol RNAi*) to knock down Perlecan from CpCs and ISCs. Perlecan was hardly detectable in the PGC region or in the boundary between the forming niche and the rest of the gonad of *tj>trol RNAi* larvae, even though Perlecan still surrounded the external perimeter of the gonad (Figure S3A). We detected no obvious alterations to ISC number or arrangement nor to the organization of the presumptive CpCs abutting the TFs (as determined by Tj$^+$ staining). These results show that the *trol* RNAi approach is an effective tool to reduce significantly Perlecan amounts in developing gonads and that a large proportion of the Perlecan found inside the gonad is secreted by *tj*$^+$ ISCs and CpCs. We then reduced Perlecan from most somatic cells of the gonad utilizing the *bab1-Gal4* line. LL3 *bab1>trol RNAi* larvae grown at 25°C (the same conditions that had previously given the CpC displacement phenotype in adult flies), possessed gonads without any obvious defects in the organization of TF and ISCs when compared to control ones (Figures 4D and 4E). However, the resolution of our analysis could be compromised by the large cellular rearrangements that take place in larval gonads.

To determine whether the displaced CpCs found in adult *bab1>trol RNAi* niches or in mosaic germaria resulted from reduced Perlecan amounts during larval/pupal gonadal development or from the loss of *trol* function in the adult, we first looked at germaria from freshly eclosed (0 to 24 h old) *bab1>trol RNAi* females grown at 25°C. We found displaced CpCs in 18.3% of experimental samples in comparison with 0% in control ones (Figures 4F and 4G). Next, we utilized the *tubulin-Gal80*^{ts} system to reduce *trol* activity only in adult niches. On this occasion, we also co-expressed the *Dicer-2* gene to enhance the RNAi phenotype. Thus, we raised *bab1-Gal4*, *tubulin-Gal80*^{ts}, *UAS-trol RNAi*, *UAS-Dicer-2* (*bab1*^{ts}*>trol RNAi + Dicer-2*) flies at 18°C till eclosion and then placed the adults at 29°C for 7 days to induce *trol RNAi* expression in the adult GSC niche. With this approach, Perlecan levels were reduced 7.5 times in experimental germaria in comparison with controls (Figure S3B). Nevertheless, we failed to observe CpC displacement in *bab1*^{ts}*>trol RNAi + Dicer-2* germaria (n = 30). Our results demonstrate that maintaining CpC rosette organization in the adult does not require high levels of Perlecan protein, and they strongly suggest that *trol* is needed during larval/pupal development for correct organization of the adult GSC niche.

trol activity regulates *D*E-cadherin levels in CpCs

Drosophila Epithelial (*D*E)-cadherin-mediated cell adhesion plays an important role during the initial stages of gonad formation in the embryo and in larval development. In the adult, *D*E-cadherin mediates CpC-GSC and EC-GSC attachment and prevents stem cell loss from the niche.[21,41–43] Considering the aberrant architecture of Perlecan mutant niches, we determined whether removal of *trol* function affected *D*E-cadherin localization. We quantified *D*E-cadherin levels at CpC-CpC and CpC-GSC boundaries in mosaic germaria containing control and *trol*$^-$ CpCs. Upon close examination of 69 boundaries from 21 germaria, we found that loss of *trol* function decreased membrane *D*E-cadherin in both CpC-CpC and CpC-GSC boundaries (68.8 ± 17.5 average fluorescence intensity in *trol*$^+$/*trol*$^+$ CpC boundaries and 46.4 ± 14.8 in *trol*$^+$/*trol*$^-$; 72.7 ± 18.9

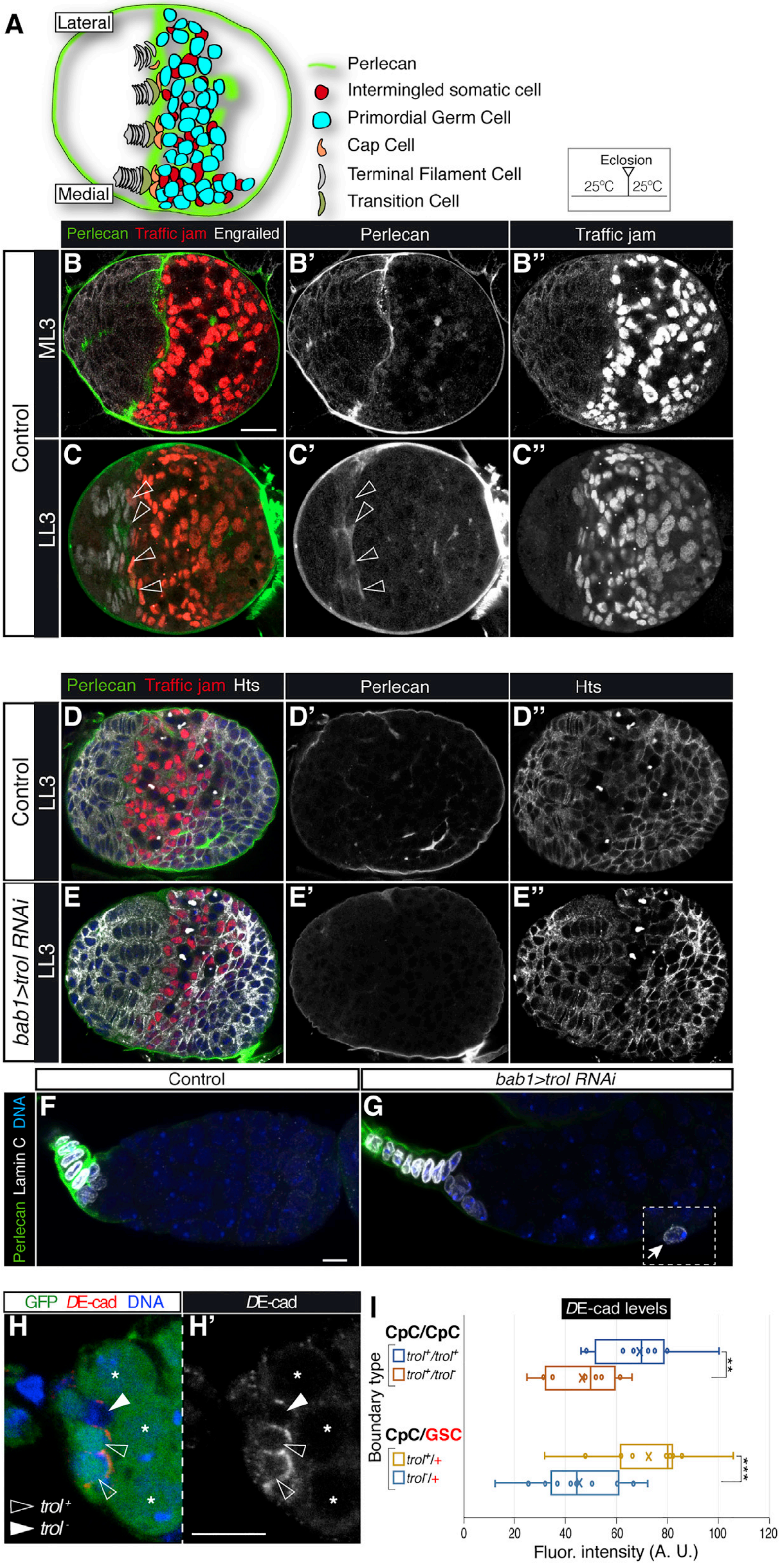

Figure 4. Perlecan expression in the larval gonad

(A) Scheme of a mid-third instar larval (ML3) gonad showing the medial-to-lateral morphogenetic wave of TF formation, the arrangement of the newly determined CpCs, and the organization of the PGCs and their associated ISCs.

(B) Z-projection of an ML3 gonad stained to visualize Perlecan and Traffic jam (to label future CpCs and ISCs). Notice the conspicuous Perlecan accumulation in the boundary region between the TFCs and the PGCs/ISCs.

(C) Z-projection of 1.5 μm of a late-third instar larval (LL3) gonad stained to visualize Perlecan, Traffic jam, and Engrailed (to label TFCs). Empty arrowheads point to accumulations of Perlecan in the forming GSC niches. LL3 gonads are found in larvae of 118–128 h of age after egg laying.

(D) Single plane of a control LL3 gonad stained to visualize Perlecan, Traffic jam, DNA, and Hts (to label spectrosomes/fusomes and the outline of most cells). The Perlecan signal in (D) appears weaker than in (C) because the latter corresponds to the projection of several z-planes.

(E) Single plane of an experimental *bab1>trol RNAi* LL3 gonad stained to visualize Perlecan, Traffic jam, DNA, and Hts. Notice the obvious decrease in Perlecan staining inside the *trol* RNAi gonad.

(F and G) Control (F) and *bab1>trol RNAi* experimental (G) germaria from freshly eclosed females stained to detect Perlecan, Lamin-C, and DNA. Images in (F, G) are z-projections of 3.15 μm of the samples; inset in (G) corresponds to a displaced CpC in a different focal plane. See Video S4 for a complete view of the experimental germarium.

(H) Z-projections of a mosaic germarium containing $trol^-$ CpCs stained to visualize *DE*-cadherin (*DE*-cad), GFP, and DNA.

(I) Quantification of *DE*-cadherin levels at $trol^+/trol^+$ or $trol^+/trol^-$ paired CpC boundaries, and at $trol^+$ CpC/$trol^+$ GSC or $trol^-$ CpC/$trol^+$ paired GSC surfaces. $trol^-$ CpCs localize significantly lower *DE*-cadherin amounts at their surfaces facing CpCs or GSCs than do $trol^+$ CpCs. Empty arrowheads, control CpCs in the rosette; solid arrowheads, mutant CpCs in the rosette; asterisks, GSCs. p values of two-tailed, paired t tests considered statistically significant between control and experimental samples are indicated (**$p \leq 0.005$; ***$p \leq 0.0005$). The mean (cross) and median (line across box) for each of the samples are shown. We quantified 10 $trol^+/trol^+$ and 11 $trol^+/trol^-$ CpC boundaries from 8 germaria and 28 $trol^+$ CpC-GSC and 20 $trol^-$ CpC-GSC boundaries from 13 germaria. Clones were induced by using the *bab1-Gal4/UASt-flp* system. Arrow, displaced CpC. Scale bars, 10 μm. See also Figure S3 and Videos S3 and S4.

in *trol*$^{+}$ CpC-GSC boundaries and 45.4 ± 17.2 in *trol*$^{-}$ CpC-GSC; Figures 4H and 4I). Analysis of mosaic LL3 gonads indicated that mutant larval TF and CpCs also displayed lower *D*E-cadherin levels than did paired controls (21.2 ± 6.1 in controls; 13.0 ± 4.9 in experimental ones; Figures S3C and S3D). Because *trol* was required in LL3 gonads and in the adult niche for proper *D*E-cadherin accumulation, we surmised that the CpC displacement phenotype was a consequence of impaired *D*E-cadherin-mediated adhesion between the mutant CpCs and other niche cells. This impaired adhesion could also explain, at least partially, the slight reduction in GSC numbers in *trol*-deficient niches (Figure 3E). To test this, we removed one copy of *shotgun,* the gene encoding for *D*E-cadherin[44] and looked at *bab1>trol RNAi* adult niches. We found that +/+; *bab1>trol RNAi* and *shotgun*/+; *bab1>trol RNAi* females displayed similar GSC numbers. However, the former showed milder CpC displacement phenotypes than did the latter (14.3% versus 19.5%, respectively; Figures S3E and S3F). Our results thus provide a direct link between loss of *trol* activity and reduced levels of a cell-cell adhesion molecule.

The finding that CpCs produce mainly the short *trol-RBB* isoform indicates a regional distribution of Perlecan variants in the niche. In addition, our mosaic analysis strongly suggests that CpCs cell-autonomously deposit interstitial Perlecan. This is in contrast to other instances in which ECM components are secreted non-autonomously by other cell types or even tissues.[22,45,46] The reason(s) for this short-range secretion could be due to a local characteristic of the interstitial matrix around CpCs that limits the range of Perlecan diffusion, or it could lie in the RBB-encoded Perlecan having different biochemical properties versus the longer isoforms. In fact, *trol-RBB* contains one small, 78-amino-acid-long exon in which 12 serine and tyrosine residues are predicted targets for O-glycosylation. This heavily glycosylated exon—present only in the short (RBB), the intermediate (RAK, but it is not expressed in the niche), and the long RBA and RAS (Figure S1A)—could confer the RBB protein biochemical properties that could explain its compartmentalized localization in the niche.

Stem cells are capable of self-renewing or to produce tissue-specific cell types. A number of factors control their behavior, including signals from nearby niche cells or the surrounding ECM.[4] Our work identifies a specialized matrix secreted by CpCs, rich in a specific Perlecan isoform and functionally relevant. This novel function of Perlecan in the formation of a proper stem cell niche could be of general importance, given the widespread presence of ECM components associated with stem cells and their niches.[5,47]

STAR★METHODS

Detailed methods are provided in the online version of this paper and include the following:

- KEY RESOURCES TABLE
- RESOURCE AVAILABILITY
  - Lead contact
  - Materials availability
  - Data and code availability
- EXPERIMENTAL MODEL AND SUBJECT DETAILS
- METHOD DETAILS
  - Fly stocks
  - Experimental genotypes
  - Immunohistochemistry
  - Imaging of fixed samples
  - Transmission Electron Microscopy (TEM)
  - Targeted DamID (TaDa)
  - DamID analysis
- QUANTIFICATION AND STATISTICAL ANALYSIS
  - Data Analysis
  - Statistical Analysis

SUPPLEMENTAL INFORMATION

Supplemental Information can be found online at https://doi.org/10.1016/j.cub.2021.01.071.

ACKNOWLEDGMENTS

A.G.-R. dedicates this work to Professor José Luis Gómez-Skarmeta, an inspirational colleague and friend, on the occasion of his untimely demise. We thank the BDSC and the VDRC for fly stocks and the DSHB (University of Iowa) for antibodies. TEM analysis was performed at the CIC, University of Granada. This work was funded by the Spanish State Research Agency (MCUI/AEI, http://www.ciencia.gob.es/; grant numbers BFU2015-65372-P, PGC2018-097115-B-I00, and MDM-2016-0687 to A.G.-R.) and by the European Regional Development Fund (http://ec.europa.eu/regional_policy/en/funding/erdf/). A.H.B. was supported by the Royal Society Darwin Trust Research Professorship and Wellcome Trust Senior Investigator Award 103792. A.H.B. acknowledges core funding to the Gurdon Institute from the Wellcome Trust (092096) and CRUK (C6946/A14492). O.J.M. was supported by an EMBO long-term fellowship (ALTF 423-2011).

AUTHOR CONTRIBUTIONS

A.D.-T., A.R.-N., J.R.P., and A.G.-R. conceived and designed research; A.D.-T., A.R.-N., J.R.P., C.S.-C.M., M.M.-M., and O.J.M. performed research; A.D.-T., O.J.M., A.H.B., and A.G.-R. analyzed data; and J.R.P. and A.G.-R. wrote the paper.

DECLARATION OF INTERESTS

The authors declare no competing interests.

Received: July 17, 2020
Revised: November 10, 2020
Accepted: January 20, 2021
Published: February 22, 2021

REFERENCES

1. Ohlstein, B., Kai, T., Decotto, E., and Spradling, A. (2004). The stem cell niche: theme and variations. Curr. Opin. Cell Biol. *16*, 693–699.
2. Schofield, R. (1978). The relationship between the spleen colony-forming cell and the haemopoietic stem cell. Blood Cells *4*, 7–25.
3. Pearson, J., López-Onieva, L., Rojas-Ríos, P., and González-Reyes, A. (2009). Recent advances in Drosophila stem cell biology. Int. J. Dev. Biol. *53*, 1329–1339.
4. Ahmed, M., and Ffrench-Constant, C. (2016). Extracellular Matrix Regulation of Stem Cell Behavior. Curr. Stem Cell Rep. *2*, 197–206.
5. Niklason, L.E. (2018). Understanding the Extracellular Matrix to Enhance Stem Cell-Based Tissue Regeneration. Cell Stem Cell *22*, 302–305.
6. Pearson, J.R., Zurita, F., Tomás-Gallardo, L., Díaz-Torres, A., Díaz de la Loza, Mdel.C., Franze, K., Martín-Bermudo, M.D., and González-Reyes,

A. (2016). ECM-regulator timp is required for stem cell niche organization and cyst production in the Drosophila ovary. PLoS Genet. *12*, e1005763.

7. Yui, S., Azzolin, L., Maimets, M., Pedersen, M.T., Fordham, R.P., Hansen, S.L., Larsen, H.L., Guiu, J., Alves, M.R.P., Rundsten, C.F., et al. (2018). YAP/TAZ-Dependent Reprogramming of Colonic Epithelium Links ECM Remodeling to Tissue Regeneration. Cell Stem Cell *22*, 35–49.e7.
8. Wang, X., and Page-McCaw, A. (2014). A matrix metalloproteinase mediates long-distance attenuation of stem cell proliferation. J. Cell Biol. *206*, 923–936.
9. Weaver, L.N., and Drummond-Barbosa, D. (2018). Maintenance of Proper Germline Stem Cell Number Requires Adipocyte Collagen in Adult *Drosophila* Females. Genetics *209*, 1155–1166.
10. O'Reilly, A.M., Lee, H.H., and Simon, M.A. (2008). Integrins control the positioning and proliferation of follicle stem cells in the Drosophila ovary. J. Cell Biol. *182*, 801–815.
11. Van De Bor, V., Zimniak, G., Papone, L., Cerezo, D., Malbouyres, M., Juan, T., Ruggiero, F., and Noselli, S. (2015). Companion Blood Cells Control Ovarian Stem Cell Niche Microenvironment and Homeostasis. Cell Rep. *13*, 546–560.
12. Yurchenco, P.D. (2011). Basement membranes: cell scaffoldings and signaling platforms. Cold Spring Harb. Perspect. Biol. *3*, a004911.
13. Farach-Carson, M.C., and Carson, D.D. (2007). Perlecan–a multifunctional extracellular proteoglycan scaffold. Glycobiology *17*, 897–905.
14. Grigorian, M., Liu, T., Banerjee, U., and Hartenstein, V. (2013). The proteoglycan Trol controls the architecture of the extracellular matrix and balances proliferation and differentiation of blood progenitors in the Drosophila lymph gland. Dev. Biol. *384*, 301–312.
15. Kamimura, K., Ueno, K., Nakagawa, J., Hamada, R., Saitoe, M., and Maeda, N. (2013). Perlecan regulates bidirectional Wnt signaling at the Drosophila neuromuscular junction. J. Cell Biol. *200*, 219–233.
16. Lindner, J.R., Hillman, P.R., Barrett, A.L., Jackson, M.C., Perry, T.L., Park, Y., and Datta, S. (2007). The Drosophila Perlecan gene trol regulates multiple signaling pathways in different developmental contexts. BMC Dev. Biol. *7*, 121.
17. Park, Y., Rangel, C., Reynolds, M.M., Caldwell, M.C., Johns, M., Nayak, M., Welsh, C.J., McDermott, S., and Datta, S. (2003). Drosophila perlecan modulates FGF and hedgehog signals to activate neural stem cell division. Dev. Biol. *253*, 247–257.
18. Voigt, A., Pflanz, R., Schäfer, U., and Jäckle, H. (2002). Perlecan participates in proliferation activation of quiescent Drosophila neuroblasts. Dev. Dyn. *224*, 403–412.
19. Xie, T., and Spradling, A.C. (2000). A niche maintaining germ line stem cells in the Drosophila ovary. Science *290*, 328–330.
20. Panchal, T., Chen, X., Alchits, E., Oh, Y., Poon, J., Kouptsova, J., Laski, F.A., and Godt, D. (2017). Specification and spatial arrangement of cells in the germline stem cell niche of the Drosophila ovary depend on the Maf transcription factor Traffic jam. PLoS Genet. *13*, e1006790.
21. Song, X., Zhu, C.H., Doan, C., and Xie, T. (2002). Germline stem cells anchored by adherens junctions in the Drosophila ovary niches. Science *296*, 1855–1857.
22. Medioni, C., and Noselli, S. (2005). Dynamics of the basement membrane in invasive epithelial clusters in Drosophila. Development *132*, 3069–3077.
23. Morin, X., Daneman, R., Zavortink, M., and Chia, W. (2001). A protein trap strategy to detect GFP-tagged proteins expressed from their endogenous loci in Drosophila. Proc. Natl. Acad. Sci. USA *98*, 15050–15055.
24. Datta, S., and Kankel, D.R. (1992). l(1)trol and l(1)devl, loci affecting the development of the adult central nervous system in Drosophila melanogaster. Genetics *130*, 523–537.
25. Friedrich, M.V., Schneider, M., Timpl, R., and Baumgartner, S. (2000). Perlecan domain V of Drosophila melanogaster. Sequence, recombinant analysis and tissue expression. Eur. J. Biochem. *267*, 3149–3159.
26. Schneider, M., Khalil, A.A., Poulton, J., Castillejo-Lopez, C., Egger-Adam, D., Wodarz, A., Deng, W.M., and Baumgartner, S. (2006). Perlecan and Dystroglycan act at the basal side of the Drosophila follicular epithelium to maintain epithelial organization. Development *133*, 3805–3815.
27. Gilboa, L., and Lehmann, R. (2004). How different is Venus from Mars? The genetics of germ-line stem cells in Drosophila females and males. Development *131*, 4895–4905.
28. Greenspan, L.J., de Cuevas, M., and Matunis, E. (2015). Genetics of gonadal stem cell renewal. Annu. Rev. Cell Dev. Biol. *31*, 291–315.
29. Southall, T.D., Gold, K.S., Egger, B., Davidson, C.M., Caygill, E.E., Marshall, O.J., and Brand, A.H. (2013). Cell-type-specific profiling of gene expression and chromatin binding without cell isolation: assaying RNA Pol II occupancy in neural stem cells. Dev. Cell *26*, 101–112.
30. Li, M.A., Alls, J.D., Avancini, R.M., Koo, K., and Godt, D. (2003). The large Maf factor Traffic Jam controls gonad morphogenesis in *Drosophila*. Nat. Cell Biol. *5*, 994–1000.
31. Decotto, E., and Spradling, A.C. (2005). The Drosophila ovarian and testis stem cell niches: similar somatic stem cells and signals. Dev. Cell *9*, 501–510.
32. Forbes, A.J., Lin, H., Ingham, P.W., and Spradling, A.C. (1996). hedgehog is required for the proliferation and specification of ovarian somatic cells prior to egg chamber formation in Drosophila. Development *122*, 1125–1135.
33. Song, X., Wong, M.D., Kawase, E., Xi, R., Ding, B.C., McCarthy, J.J., and Xie, T. (2004). Bmp signals from niche cells directly repress transcription of a differentiation-promoting gene, bag of marbles, in germline stem cells in the Drosophila ovary. Development *131*, 1353–1364.
34. Xie, T., and Spradling, A.C. (1998). decapentaplegic is essential for the maintenance and division of germline stem cells in the Drosophila ovary. Cell *94*, 251–260.
35. Zhang, Y., and Kalderon, D. (2001). Hedgehog acts as a somatic stem cell factor in the Drosophila ovary. Nature *410*, 599–604.
36. Tazuke, S.I., Schulz, C., Gilboa, L., Fogarty, M., Mahowald, A.P., Guichet, A., Ephrussi, A., Wood, C.G., Lehmann, R., and Fuller, M.T. (2002). A germline-specific gap junction protein required for survival of differentiating early germ cells. Development *129*, 2529–2539.
37. López-Onieva, L., Fernández-Miñán, A., and González-Reyes, A. (2008). Jak/Stat signalling in niche support cells regulates dpp transcription to control germline stem cell maintenance in the Drosophila ovary. Development *135*, 533–540.
38. Dansereau, D.A., and Lasko, P. (2008). The development of germline stem cells in Drosophila. Methods Mol. Biol. *450*, 3–26.
39. Yatsenko, A.S., and Shcherbata, H.R. (2018). Stereotypical architecture of the stem cell niche is spatiotemporally established by miR-125-dependent coordination of Notch and steroid signaling. Development *145*, dev159178.
40. Sahut-Barnola, I., Godt, D., Laski, F.A., and Couderc, J.-L. (1995). Drosophila ovary morphogenesis: analysis of terminal filament formation and identification of a gene required for this process. Dev. Biol. *170*, 127–135.
41. Jenkins, A.B., McCaffery, J.M., and Van Doren, M. (2003). Drosophila E-cadherin is essential for proper germ cell-soma interaction during gonad morphogenesis. Development *130*, 4417–4426.
42. Lai, C.M., Lin, K.Y., Kao, S.H., Chen, Y.N., Huang, F., and Hsu, H.J. (2017). Hedgehog signaling establishes precursors for germline stem cell niches by regulating cell adhesion. J. Cell Biol. *216*, 1439–1453.
43. Wang, X., and Page-McCaw, A. (2018). Wnt6 maintains anterior escort cells as an integral component of the germline stem cell niche. Development *145*, dev158527.
44. Tepass, U., Gruszynski-DeFeo, E., Haag, T.A., Omatyar, L., Török, T., and Hartenstein, V. (1996). shotgun encodes Drosophila E-cadherin and is preferentially required during cell rearrangement in the neurectoderm and other morphogenetically active epithelia. Genes Dev. *10*, 672–685.
45. Sánchez-Sánchez, B.J., Urbano, J.M., Comber, K., Dragu, A., Wood, W., Stramer, B., and Martín-Bermudo, M.D. (2017). Drosophila Embryonic

CellPress
OPEN ACCESS

Hemocytes Produce Laminins to Strengthen Migratory Response. Cell Rep. *21*, 1461–1470.

46. Pastor-Pareja, J.C., and Xu, T. (2011). Shaping cells and organs in Drosophila by opposing roles of fat body-secreted Collagen IV and perlecan. Dev. Cell *21*, 245–256.
47. Watt, F.M., and Huck, W.T. (2013). Role of the extracellular matrix in regulating stem cell fate. Nat. Rev. Mol. Cell Biol. *14*, 467–473.
48. Wolfstetter, G., Shirinian, M., Stute, C., Grabbe, C., Hummel, T., Baumgartner, S., Palmer, R.H., and Holz, A. (2009). Fusion of circular and longitudinal muscles in Drosophila is independent of the endoderm but further visceral muscle differentiation requires a close contact between mesoderm and endoderm. Mech. Dev. *126*, 721–736.
49. Kumagai, C., Kadowaki, T., and Kitagawa, Y. (1997). Disulfide-bonding between Drosophila laminin β and γ chains is essential for α chain to form α betagamma trimer. FEBS Lett. *412*, 211–216.
50. Lobo-Pecellín, M., Marín-Menguiano, M., and González-Reyes, A. (2019). *mastermind* regulates niche ageing independently of the *Notch* pathway in the *Drosophila* ovary. Open Biol. *9*, 190127.
51. Gunawan, F., Arandjelovic, M., and Godt, D. (2013). The Maf factor Traffic jam both enables and inhibits collective cell migration in Drosophila oogenesis. Development *140*, 2808–2817.
52. Guo, Z., and Wang, Z. (2009). The glypican Dally is required in the niche for the maintenance of germline stem cells and short-range BMP signaling in the Drosophila ovary. Development *136*, 3627–3635.
53. Bolívar, J., Pearson, J., López-Onieva, L., and González-Reyes, A. (2006). Genetic dissection of a stem cell niche: the case of the *Drosophila* ovary. Dev. Dyn. *235*, 2969–2979.
54. Andersen, D., and Horne-Badovinac, S. (2016). Influence of ovarian muscle contraction and oocyte growth on egg chamber elongation in Drosophila. Development *143*, 1375–1387.
55. Chou, T.-B., and Perrimon, N. (1992). Use of a yeast site-specific recombinase to produce female germline chimeras in Drosophila. Genetics *131*, 643–653.
56. Valencia-Expósito, A., Grosheva, I., Míguez, D.G., González-Reyes, A., and Martín-Bermudo, M.D. (2016). Myosin light-chain phosphatase regulates basal actomyosin oscillations during morphogenesis. Nat. Commun. *7*, 10746.
57. Maimon, I., and Gilboa, L. (2011). Dissection and staining of Drosophila larval ovaries. J. Vis. Exp. (51), 2537.
58. Timpl, R. (1982). Antibodies to collagens and procollagens. Methods Enzymol. *82* (Pt A), 472–498.
59. Reynolds, E.S. (1963). The use of lead citrate at high pH as an electron-opaque stain in electron microscopy. J. Cell Biol. *17*, 208–212.
60. Marshall, O.J., Southall, T.D., Cheetham, S.W., and Brand, A.H. (2016). Cell-type-specific profiling of protein-DNA interactions without cell isolation using targeted DamID with next-generation sequencing. Nat. Protoc. *11*, 1586–1598.
61. Marshall, O.J., and Brand, A.H. (2015). damidseq_pipeline: an automated pipeline for processing DamID sequencing datasets. Bioinformatics *31*, 3371–3373.
62. Ramírez, F., Bhardwaj, V., Arrigoni, L., Lam, K.C., Grüning, B.A., Villaveces, J., Habermann, B., Akhtar, A., and Manke, T. (2018). High-resolution TADs reveal DNA sequences underlying genome organization in flies. Nat. Commun. *9*, 189.
63. López-Delisle, L., Rabbani, L., Wolff, J., Bhardwaj, V., Backofen, R., Grüning, B., Ramírez, F., and Manke, T. (2020). pyGenomeTracks: reproducible plots for multivariate genomic data sets. Bioinformatics, btaa692.

STAR★METHODS

KEY RESOURCES TABLE

REAGENT or RESOURCE	SOURCE	IDENTIFIER
Antibodies		
mouse anti-Hts	Developmental Studies Hybridoma Bank	Cat# 1B1; RRID: AB_528070
mouse anti-Lamin C	Developmental Studies Hybridoma Bank	Cat# LC28.26; RRID: AB_528339
rabbit anti-Vasa	Prof. Ruth Lehmann	N/A
rat anti-*DE*-cadherin	Developmental Studies Hybridoma Bank	Cat# DCAD2; RRID: AB_528120
mouse anti-Engrailed	Developmental Studies Hybridoma Bank	Cat# 4D9; RRID: AB_528224
goat anti-GFP, FITC-conjugated	Abcam	Cat# ab6662; RRID: AB_305635
rabbit anti-Nidogen	Wolfstetter et al.[48]	N/A
rabbit anti-Laminin β1	Kumagai et al.[49]	N/A
guinea pig anti-Hedgehog	Lobo-Pecellín et al.[50]	N/A
guinea pig anti-Traffic jam	Gunawan et al.[51]	N/A
rabbit anti-Perlecan	This work	N/A
Chemicals		
VECTASHIELD	Vector Laboratories	Cat# H1000; RRID: AB_2336789
PBS tablets	Sigma-Aldrich	Cat# P4417
Tween 20	Sigma-Aldrich	Cat# P9416
Rhodamine-phalloidin	Biotium	Cat# BT-00027
BSA	Sigma-Aldrich	Cat# 05470
FBS	Sigma-Aldrich	Cat# F2442
Triton x-100	Sigma-Aldrich	Cat# T8787
Glutaraldehyde	Sigma-Aldrich	Cat# G5882
EMbed 812	Electron Microscopy Sciences	Cat# 14120
EDTA	Sigma-Aldrich	Cat# E6758
QIAamp DNA Micro Kit	Qiagen	Cat# 56304
DpnI and Cut Smart buffer	NEB	Cat# R0176S
PCR Purification kit	Qiagen	Cat# 28104
Tris	Sigma-Aldrich	Cat# T2694
DpnII and DpnII buffer	NEB	Cat# R0543S
Advantage 2 cDNA polymerase	Clontech	Cat# 639201
AlwI	NEB	Cat# R0513S
RNase A (DNase free)	Roche	Cat# 11119915001
Qubit assay tubes	Invitrogen	Cat# Q32856
Qubit dsDNA HS assay kit	Invitrogen	Cat# Q32851
Genomic DNA ScreenTape	Agilent	Cat# 5067-5365
Reagents for TapeStation	Agilent	Cat# 5067-5366
Reagents for Bioanalyzer: DNA 1000 kit	Agilent	Cat# 5067-1504
Agencourt AMPure XP Beads	Beckman Coulter	Cat# A63880
Quick ligation kit	NEB	Cat# M2200S
T4 ligase (400,000 U/ml)	NEB	Cat# M0202S
T4 DNA polymerase	NEB	Cat# M0203S
Klenow fragment	NEB	Cat# M0210S
Klenow 3’-5’exo-enzyme	NEB	Cat# M0212S
T4 polynucleotide kinase	NEB	Cat# M0201S

(*Continued on next page*)

Continued

REAGENT or RESOURCE	SOURCE	IDENTIFIER
NEBNext High-Fidelity 2x PCR Master Mix	NEB	Cat# M0541S
dNTPs	NEB	Cat# N0446S
Experimental Models: Organisms/Strains		
y w	Laboratory stock	RRID: BDSC_1495
trol^{null}	Voigt et al.[18]	N/A
shg^{1}	Tepass et al.[44]	N/A
UASt-trol RNAi	VDRC	22642
UASt-sh:GFP RNAi	VDRC	313432
en-Gal4	Guo and Wang[52]	RRID: BDSC_30564
bab1-Gal4	Bolívar et al.[53]	N/A
tj-Gal4	Guo and Wang[52]	N/A
UASt-LT3-Dam, *UASt-LT3-Dam PolII*	Southall et al.[29]	N/A
vkg::GFP	Medioni et al.[22] and Morin et al.[23]	N/A
trol::GFP	Morin et al.[23]	N/A
tub-Gal80^{ts}	BDSC	RRID: BDSC_7108
y w, hs-flp12 pUbi-nls GFP FRT-101	Laboratory stock	N/A
UASt-FLP	Laboratory stock	N/A
Software and Algorithms		
Fiji	Open Source	https://fiji.sc/
Imaris	Oxford Instruments	https://imaris.oxinst.com/
Deposited Data		
Raw sequencing data	This study	GEO accession number: GSE164866

RESOURCE AVAILABILITY

Lead contact

Further information and requests for resources and reagents should be directed to and will be fulfilled by the Lead Contact, Acaimo González-Reyes (agonrey@upo.es).

Materials availability

Fly lines generated in this study and the anti-Perlecan antibody are available without restrictions from the Lead Contact.

Data and code availability

Original DamID sequencing data have been deposited to the Gene Expression Omnibus website (https://www.ncbi.nlm.nih.gov/geo/; accession number GEO: GSE164866).

EXPERIMENTAL MODEL AND SUBJECT DETAILS

Fruit flies *D. melanogaster* were reared on standard wheat flour-agar medium or on the richer Nutri-Fly™ "German Food" Sick Fly Formulation (Genesee Scientific). Flies were grown at 25°C with relative humidity of approx. 50% and a 12h dark/12h light cycle, unless otherwise noted. For strain details see the Key Resource Table.

METHOD DETAILS

Fly stocks

The *traffic jam-Gal4* driver (*tj-Gal4*) is expressed in most of the somatic cells of the ovary, including the muscle sheath.[20,30,54] *bric-a-brac 1-Gal4* (*bab1-Gal4*) is expressed at higher levels in TFCs and CpCs, and weakly in ECs and in few germarial follicle cells.[53] *engrailed-Gal4* (*en-Gal4*) is expressed in TFCs and CpCs.[52]

For loss-of-function experiments, we either used RNA interference or induced somatic clones utilising the FRT/FLP technique. The RNAi knockdown was performed using the Gal4-UAS system. To knock-down *trol* RNA levels, flies of the appropriate genotype were either grown and kept at 25°C or, when harbouring the *tub-Gal80*^{ts} construct, grown at 18°C and shifted from 18°C to 29°C for one

week upon hatching and prior to dissection. The RNAi construct used should target all known *trol* isoforms (Figure S1A). To induce somatic clones with the FRT/FLP technique,[55] we generated either *trol^{null}* FRT-101/*hs-flp12 ubi-nlsGFP* FRT-101 or *trol^{null}* FRT-101/*hs-flp12 ubi-nlsGFP* FRT-101;; *bab1-Gal4 UASt-flp*/TM2 females. In the former genotype, recombination between the FRT-101 sites was induced by the activation of the *hs-flp* transgene after transferring adult females or larvae to 37°C for 1 hour. In the latter, recombination was achieved by the *bab1-Gal4*-mediated expression of *UASt-flp* or, when indicated, by both, *bab1-Gal4/UASt-flp* and by heat-shock. Mutant clones were marked by the absence of GFP. *trol^{null}* is a deletion of the entire gene that eliminates all known *trol* isoforms.[18] In RNAi experiments, *bab1-Gal4* or *tj-Gal4* were combined with the corresponding *UASt-RNAi* line.

Experimental genotypes

Figure 1

(B) *w*; *viking::GFP*
(C, D, F, G, H) *y w*
(E) *w; trol::GFP*

Figures 3

(A-E) control: *w, UASt-trol RNAi/+;; TM6B/+*
bab1>trol RNAi: w, UASt-trol RNAi/+;; bab1-Gal4/+
(F, G) *w, trol^{null} FRT-101/y w, hs-FLP12 pUbi-nls GFP FRT-101; bab1-Gal4, UASt-FLP/+*

Figure 4

(B, C) *y w*
(D-G) control*: w, UASt-trol RNAi/+;; TM6B/+*
bab1>trol RNAi: w, UASt-trol RNAi/+;; bab1-Gal4/+
(H) *w, trol^{null} FRT-101/y w, hs-FLP12 pUbi-nls GFP FRT-101; bab1-Gal4, UASt-FLP/+*

Figure S1

(C) *y w*

Figure S2

(A-D) *w, trol^{null} FRT-101/y w, hs-flp12 pUbi-nls GFP FRT-101; bab1-Gal4, UASt-FLP/+*
(E) control: *trol::GFP;; UAS-sh:GFP/TM2*
trol::GFP;; bab1>GFP RNAi: trol::GFP;; bab1-Gal4/UAS-sh:GFP

Figure S3

(A) *tj>trol RNAi: w, UASt-trol RNAi/+; tj-Gal4/+*
(B) control: *w, UASt-trol RNAi/+;; TM6B/+*
bab1^{ts}>trol RNAi+Dicer-2: w, UASt-trol RNAi/+; tub-Gal80^{ts}/+; bab1-Gal4/UAS-Dicer-2
(C) *w, trol^{null} FRT-101/y w, hs-flp12 pUbi-nls GFP FRT-101*
(E, F) control +/+: *w, UASt-trol RNAi/+; Gla/+; TM6B/+*
control *shg*/+: *w, UASt-trol RNAi/+; shg^{1}/+; TM6B/+*
bab1>trol RNAi: w, UASt-trol RNAi/+; Gla/+; bab1-Gal4/+
shg/+; bab1>trol RNAi: w, UASt-trol RNAi/+; shg^{1}/+; bab1-Gal4/+

Video S1

bab1>trol RNAi: w, UASt-trol RNAi/+;; bab1-Gal4/+

Video S2

w, trol^{null} FRT-101/y w, hs-FLP12 pUbi-nls GFP FRT-101; bab1-Gal4, UASt-FLP/+

Video S3

control: w, UASt-trol RNAi/+; +/CyO
w; trol::GFP

Video S4

bab1>trol RNAi: w, UASt-trol RNAi/+;; bab1-Gal4/+

Immunohistochemistry

Adult flies were yeasted for 2 days before dissection in PBT (PBS + 0.1% Tween 20). Ovary stainings were performed at room temperature as described in.[56] Chemical dyes were added after antibody incubation. To visualise actin filaments, samples were incubated 20 minutes in PBT + 1:20 Rhodamine-phalloidin. To detect DNA, samples were incubated for 10 minutes in PBT + Hoechst (Sigma, 5mg/ml; used 1:1000).

To stain third instar larval gonads, dissected gonads embedded in larval fat body were incubated in 5% formaldehyde in Ringer's medium for 20 minutes and then washed for 5, 10 and 45 minutes in 1% PBT (PBT + 1% BSA).[57] Samples were blocked with 0.3% PBTB (0.3% Triton X-100 and 1% BSA in PBS) for one hour with gentle agitation and incubated with the primary antibody diluted in 0.3% PBTB overnight at 4°C with agitation. Next day, samples were washed three times in 0.3% PBTB and blocked with 0.3% PBTB supplemented with 5% foetal bovine serum (FBS, Sigma) for 1 hour. After blocking, samples were incubated with the secondary antibodies in blocking solution for 2 hours. Samples were washed three times in 0.3% PBT and mounted in VECTASHIELD (Vector Laboratories).

Primary antibodies used were: Mouse monoclonal anti-Hts (Developmental Studies Hybridoma Bank, DSHB), 1:100; Mouse monoclonal anti-Lamin C (DSHB), 1:100; Rabbit anti-Vasa (a gift from R. Lehmann), 1:2000; Rat anti-*D*E-cadherin, DCAD2 (DSHB), 1:100; Mouse monoclonal anti-Engrailed, 4D9 (DSHB), 1:10; Goat anti-GFP, FITC-conjugated (Abcam, ab6662), 1:500; Rabbit anti-Nidogen,[48] 1:100; Rabbit anti-Laminin β1,[49] 1:1000; Guinea pig anti-Hedgehog,[50] 1:500; Guinea pig anti-Traffic Jam (a gift from D. Godt),[51] 1:5000. The anti-Perlecan antibody was raised by ProteoGenix SAS (France) following a protocol based on.[25] In short, a 2310bp cDNA coding for Domain V of the Perlecan protein was codon optimised for its expression in mammalian cells and ligated into an episomal expression vector. The vector was transfected into human 293-EBNA cells (Invitrogen) and serum-free medium was collected for protein purification. Antibodies were obtained after Ni-affinity purification followed by size-exclusion chromatography. Immunisation of rabbits and affinity-purification of antibodies followed standard protocols.[58] The affinity-purified antibody was used at a concentration of 1:2000. Antibody specificity was demonstrated by the lack of Perlecan staining in CpCs homozygous for a protein-null mutation in the *trol* gene and by the strong reduction in Perlecan levels upon *trol RNAi* knock-down (see main text). Secondary antibodies FITC, Cy2, Cy3 and Cy5 (Jackson Immuno Research Laboratories, Inc.) were used at 1:100.

Imaging of fixed samples

Images were acquired with a Leica SP5 confocal microscope, analysed utilising Imaris and ImageJ, and processed with Adobe Photoshop and Adobe Illustrator. 3-D images of fixed samples were taken with a 40x/1.3 NA or 63x/1.4 NA oil immersion objectives.

Transmission Electron Microscopy (TEM)

TEM samples were prepared following standard procedures. Briefly, ovaries were dissected in PBS + 0.1% Tween-20 and fixed for 2 hours at 4°C in 3% glutaraldehyde/l% paraformaldehyde (vol./vol.) in 0.05 M cacodylate buffer (pH 7.4). After three 10 min. washes in cacodylate buffer 0.1 M at 4°C, ovaries were postfixed for 1 hour at 4°C in the dark (1% OsO_4, 1% $K_3Fe[CN]_6$ in water) and rinsed three times in distilled water at 4°C and stained for 2 hours at room temperature (RT) in darkness (0.5% uranyl acetate). Next, ovaries were rinsed in distilled water and dehydrated through an ethanol rising series (50%, 70%, 90% and 3x100%; 10 min. each) at RT. Ovaries were then infiltrated with Embed 812 resin (Electron Microscopy Sciences) as follows: EMbed 812/ethanol 100%. 1:2, 1:1 and 2:1 for 1 hour at RT each, and in EMbed 812 overnight at 4°C. The resin-embedded specimens were polymerised by incubation in fresh EMbed 812 during 48 hours at 60°C in flat plastic embedding molds. The inclusion blocks were cut in 50-70 nm thick sections with a DIATOME diamond-blade fixed on a Reichert Jung Ultramicrotome and mounted on copper grids. Sections were counterstained with 1% uranyl acetate in 50% ethanol for 1 min. and then stained with lead citrate for 5 min. in a CO_2-free atmosphere.[59] Sections were examined with a Zeiss EM902 electron microscope at 80Kv, and photographed at 50.000x magnification.

Targeted DamID (TaDa)

The Targeted DamID (TaDa) technique is a variation of DNA adenine methyltransferase identification (DamID). The TaDa approach assesses genome-wide protein binding *in vivo* in a cell type-specific manner but without the need for cell isolation or purification. In short, TaDa utilises the Gal4/UAS system to express a fusion of the Dam methylase and the RNA polymerase II core subunit RpII215 (Dam-Pol II) in specific cell types. Dam-Pol II in turn tags interacting chromatin by methylating adenines within GATC sequences. RNA Pol II occupancy can then be identified upon digestion of isolated genomic DNA with the methylation-sensitive DpnI enzyme. Subsequent sequencing of the digested DNA fragments allows the profiling of RNA Pol II occupancy in cells of interest.[29]

Flies carrying the *UASt-LT3-Dam tub-Gal80^{ts}* or the *UASt-LT3-DamPolII tub-Gal80^{ts}* systems[29] were crossed to *en-Gal4*, *bab1-Gal4* or *tj-Gal4* and reared at 18°C. After hatching, adults were placed at 29°C for 24 hours to induce Dam-PolII or Dam expression. Genomic DNA was extracted from 150 dissected ovaries per replicate (Qiagen DNeasy kit, 69181) and methylated DNA processed and amplified as described.[60] Briefly, genomic DNA was digested overnight with DpnI (NEB) (which cuts methylated GATC sequences) and adaptor sequences ligated to the cut DNA fragments. Following a subsequent digestion with DpnII (NEB) (which selectively cuts at unmethylated GATC sites), fragments with consecutive methylated GATCs were amplified via PCR using primers specific to the ligated adaptors using Advantage cDNA polymerase (Clontech).

DamID samples were prepared for next-generation sequencing as previously described.[60] Briefly, DNA was sonicated using a Bioruptor Plus (Diagenode) to an average fragment size of 300bp and DamID adaptors were removed through digestion with Sau3AI,

before end-repair, A-tailing, Illumina adaptor ligation, and PCR amplification. 50bp single-end reads were obtained via a HiSeq 1500 (Illumina).

We processed and sequenced two biological replicates for the *tj-GAL4* driver, and one replicate each of the *en-Gal4* and *bab1-GAL4* drivers. Results are listed in Data S1.

DamID analysis

Illumina NGS reads were aligned back to the Dm6 reference genome and enrichment profiles calculated using damidseq_pipeline with default settings,[61] and replicates were scaled and averaged. Pol II occupancy figures were generated using pyGenomeTracks.[62,63] Pol II occupancy across gene bodies was determined using polii.gene.call[60] with genes considered to have significant Pol II occupancy at FDR<0.01.

QUANTIFICATION AND STATISTICAL ANALYSIS

Data Analysis

To quantify fluorescent signal in control and experimental samples images were captured using identical confocal settings. Z-sections were taken every 0.5 μm. Colour depth was set to 8-bit and configured so that most pixels were within the range of detection. Fluorescent intensities of the FITC, GFP or Cyanine markers used were quantified in the CpC and TF region of the niche by drawing small boxes. When appropriate, paired comparisons of cells and/or cell boundaries of the same germarium or larval gonad were done. Quantification was performed only in germaria lacking the muscle sheath. Image stacks were pre-processed using the standard background subtraction function of ImageJ. For quantifications, we utilised the "Measurement points" tool of the IMARIS software and/or the "ROI measurement" tool from ImageJ.

Statistical Analysis

Experiments were performed with at least three biological replicas. Germaria were collected from at least 5 different adult females grown under equivalent environmental conditions. The average values ± standard deviations are represented. P-values were obtained using a Student's t-test to determine values that were significantly different (*: P£0.05, **: P£0.005, ***: P£0.0005). Numbers in Figures 3B–3F, S2E and S3B, S3E and S3F refer to number of germaria analysed (n).

Current Biology, Volume *31*

Supplemental Information

Stem cell niche organization in the *Drosophila* ovary requires the ECM component Perlecan

Alfonsa Díaz-Torres, Alicia E. Rosales-Nieves, John R. Pearson, Carmen Santa-Cruz Mateos, Miriam Marín-Menguiano, Owen J. Marshall, Andrea H. Brand, and Acaimo González-Reyes

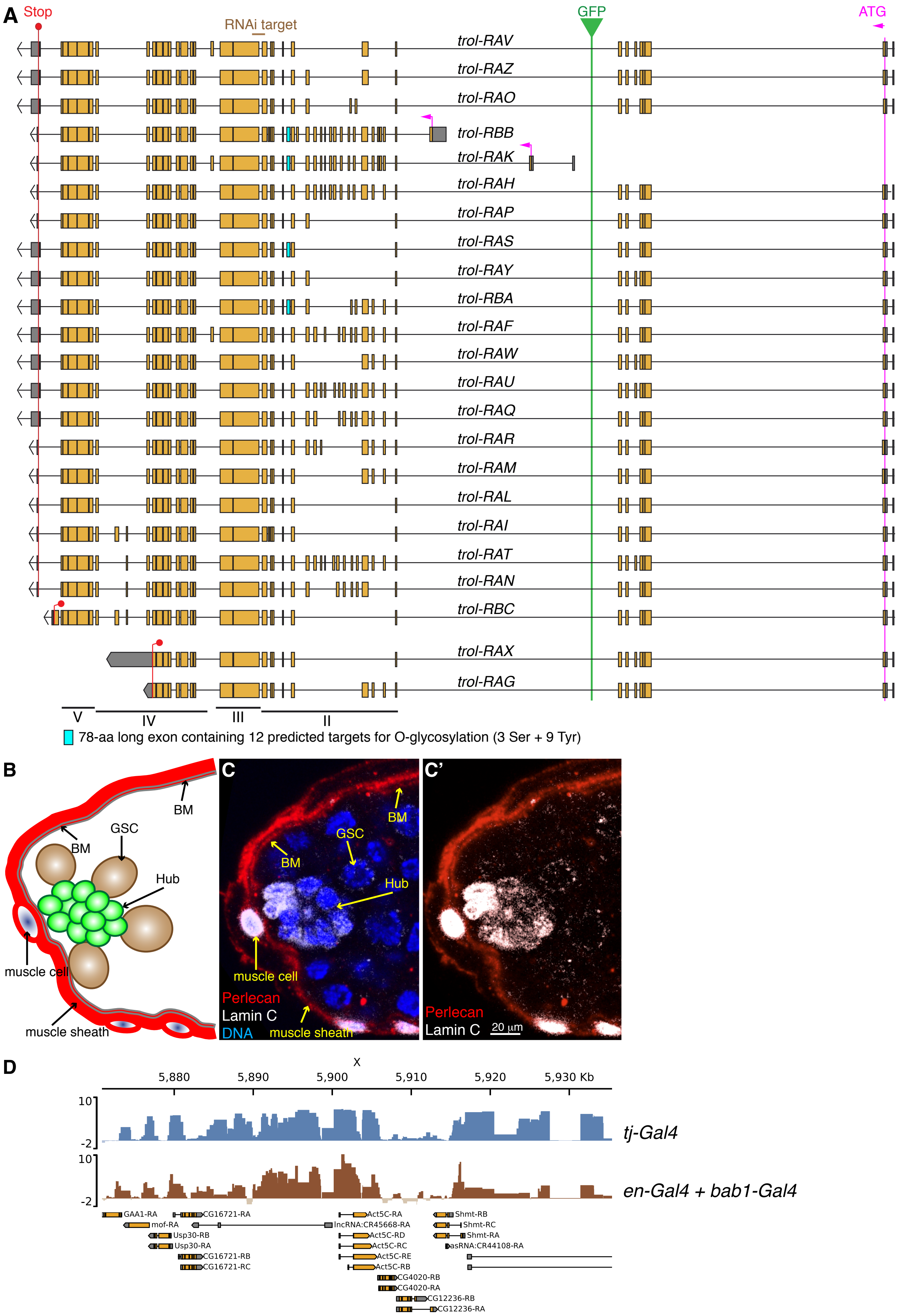
A
Stop
RNAi target
GFP
ATG
trol-RAV
trol-RAZ
trol-RAO
trol-RBB
trol-RAK
trol-RAH
trol-RAP
trol-RAS
trol-RAY
trol-RBA
trol-RAF
trol-RAW
trol-RAU
trol-RAQ
trol-RAR
trol-RAM
trol-RAL
trol-RAI
trol-RAT
trol-RAN
trol-RBC
trol-RAX
trol-RAG
V
IV
III
II
78-aa long exon containing 12 predicted targets for O-glycosylation (3 Ser + 9 Tyr)
B
BM
GSC
BM
Hub
muscle cell
muscle sheath
C
BM
GSC
BM
Hub
muscle cell
Perlecan
Lamin C
DNA
muscle sheath
C'
Perlecan
Lamin C
20 μm
D
X
5,880
5,890
5,900
5,910
5,920
5,930 Kb
10
-2
tj-Gal4
10
-2
en-Gal4 + bab1-Gal4
GAA1-RA
CG16721-RA
mof-RA
Usp30-RB
Usp30-RA
CG16721-RB
CG16721-RC
Act5C-RA
lncRNA:CR45668-RA
Act5C-RD
Act5C-RC
Act5C-RE
Act5C-RB
CG4020-RB
CG4020-RA
CG12236-RB
CG12236-RA
Shmt-RB
Shmt-RC
Shmt-RA
asRNA:CR44108-RA

Figure S1: Organisation of the *trol* gene. Perlecan accumulation in the male GSC niche. Transcription of the *Actin 5c (Act5c)* gene in different somatic cell types of the adult ovary. Related to Figures 1 and 2. (A) According to JBrowse (FlyBase2020_04), the *trol* gene can give rise to 23 different predicted isoforms. The ATGs corresponding to the long, intermediate and short versions, the STOP codons, the target sequence for the *trol RNAi* construct and the insertion point of the GFP exon are shown for all isoforms. Only the large isoforms are expected to incorporate GFP. The Perlecan antibody used in this study was raised against domain V of the protein. *Drosophila trol* lacks Domain I of the human orthologue gene. Exon in light blue is specific for only four isoforms and it is predicted to be O-glycosylated in 12 out of 78 residues. The RNAi target is shown in light brown. All isoforms should be targets of the interference construct. **(B)** Schematic representation of the *Drosophila* testis GSC niche. The anterior tip is home to the hub and a number of male GSCs attached to the hub cells. Each GSC is flanked by two somatic stem cells (cyst stem cells; not shown). The niche is surrounded by a basement membrane (BM) and an external muscle sheath. **(C)** Confocal image showing the tip of a *Drosophila* testis. Hub cells accumulate Lamin C (white) in a similar manner to CpCs. However, Perlecan (red) is not detected in the hub, even though it accumulates in the BM and the muscle sheath. Muscle cells also express nuclear Lamin C. DNA is shown in blue (Hoechst staining). **(D)** Cell-type-specific profiling of *Act5c* expression in TF cells, CpCs and ECs (*en-Gal4 + bab1-Gal4*) or in most of the somatic cells of the adult ovary (*tj-Gal4*) using the TaDa technique. Scale bars represent log2 ratio change between Dam-Pol II and Dam (reference) samples. The data are scaled so that the Pol II occupancy between the two different groups of cell types should be equivalent. Scale bar= 20µm.

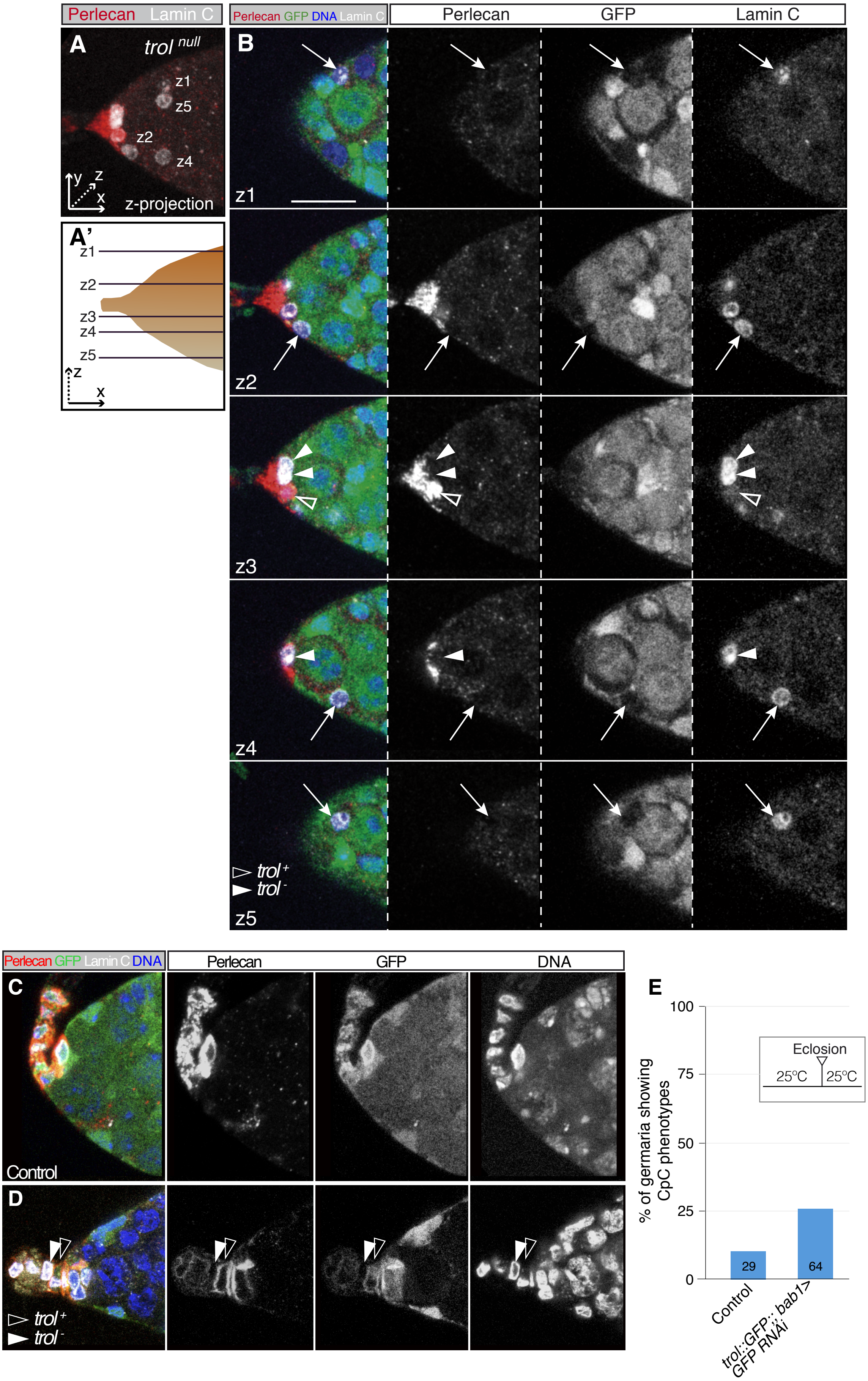

Perlecan Lamin C
A
trol null
z1
z5
z2
z4
y z x
z-projection
A'
z1
z2
z3
z4
z5
z
x
Perlecan GFP DNA Lamin C
Perlecan
GFP
Lamin C
B
z1
z2
z3
z4
z5
trol+
trol-
Perlecan GFP Lamin C DNA
Perlecan
GFP
DNA
C
Control
D
trol+
trol-
E
% of germaria showing CpC phenotypes
100
75
50
25
0
Eclosion
25ºC
25ºC
29
64
Control
trol::GFP;; bab1> GFP RNAi

Figure S2: Loss of *trol* activity induces cap cell displacement. Related to Figure 3. (A) Z-projection (top view) of a mosaic germarium containing several *trol* mutant CpCs. At least four of the *trol*$^-$ cells are not associated with the CpC rosette found at the base of the terminal filament. **(A')** Representation showing the different z-planes used in this analysis (lateral view). **(B)** Individual optical sections displaying the spatial arrangement of the mutant cells with respect to the anterior CpC rosette. CpCs are labelled with Lamin C. *trol* mutant cells are marked by the lack of GFP and Perlecan staining. Plane z3 shows a *trol*$^+$ CpC with high Perlecan levels. Its Lamin-C staining can be observed better in plane z2. **(C)** Z-sections of a control and **(D)** a mosaic germarium containing a *trol*$^-$ TF cell stained to visualise Perlecan, GFP, Lamin C and DNA. The mutant TF cell (GFP$^-$; solid arrowhead) shows a noticeable reduction in Perlecan levels. **(E)** Quantification of the number of abnormal niches containing displaced CpCs in control and in experimental (*trol::GFP;; bab1>GFP RNAi*) germaria grown at 25°C. Numbers in bars refer to number of germaria analysed. Arrows: displaced *trol*$^-$ CpCs; empty arrowheads: control CpCs in the rosette; solid arrowheads: mutant CpCs in the rosette. (A, B) Clones were induced using the *bab1-Gal4/UASt-flp* system. (C) To increase the occurrence of mitotic recombination, in addition to inducing *flp* expression with the *bab1-Gal4/UASt-flp* system, flies of the appropriate genotype were subjected to heat-shock to express *flp* from the *hs-flp* construct (see STAR METHODS in the main text). Scale bar = 20µm.

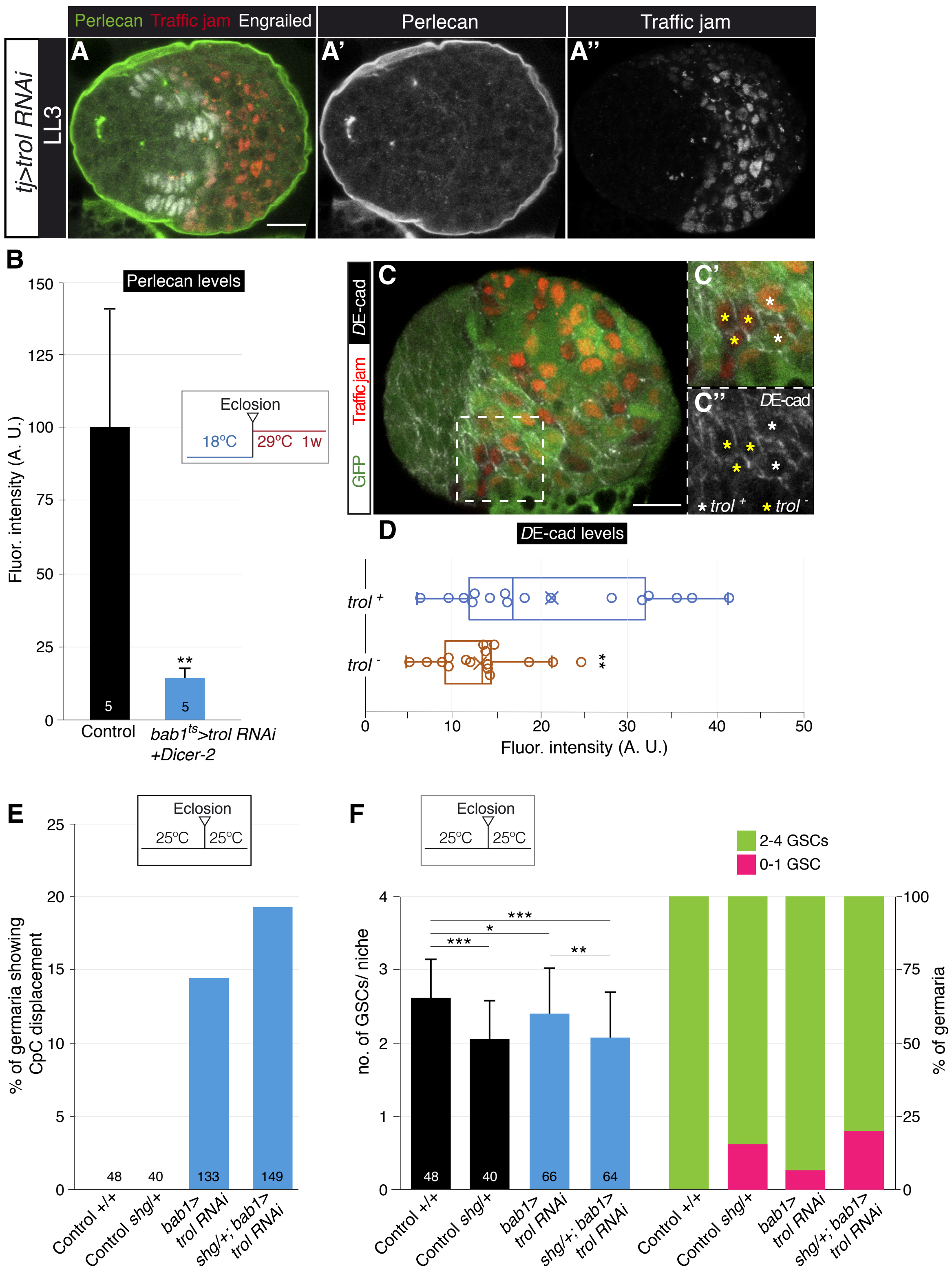

Perlecan Traffic jam Engrailed
Perlecan
Traffic jam
tj>trol RNAi
LL3
A
A'
A''
B
Perlecan levels
Fluor. intensity (A. U.)
Eclosion
18ºC
29ºC 1w
Control
bab1ts>trol RNAi +Dicer-2
C
C'
C''
DE-cad
Traffic jam
GFP
* trol +
* trol -
D
DE-cad levels
trol +
trol -
Fluor. intensity (A. U.)
E
Eclosion
25ºC
25ºC
% of germaria showing CpC displacement
Control +/+
Control shg/+
bab1> trol RNAi
shg/+; bab1> trol RNAi
F
2-4 GSCs
0-1 GSC
no. of GSCs/ niche
% of germaria

Figure S3: Low Perlecan levels in *tj>trol RNAi* gonads. Quantification of Perlecan levels in *bab1^{ts}>trol RNAi+dicer* niches. Quantification of *D*E-cadherin levels in mosaic gonads. Genetic interaction between *shotgun* and *trol* in adult niches. Related to Figure 4. (A) Z-projection of a *tj>trol RNAi* LL3 gonad stained to visualise Perlecan, Traffic jam and Engrailed (to label TF cells). As an indication of the effectiveness of the *trol* RNAi tool, notice the strong reduction in Perlecan levels at the base of the TFs and in the PGC/ISC area. This experimental gonad corresponds to the control shown in Figure 4C. **(B)** Quantification of anti-Perlecan immunofluorescence signal in control and *bab1^{ts}>trol RNAi + Dicer-2* germaria grown at 18°C and kept for 1 week at 29°C upon eclosion. **(C)** Z-projection of a mosaic LL3 gonad stained to visualise Traffic jam, GFP and *D*E-cadherin. In this particular example, three mutant Tj-positive cells at the anterior limit of the germline cluster (considered to be prospective CpCs; yellow asterisks) are compared to neighbour control cells (white asterisks). **(D)** Quantification of *D*E-cadherin levels at *trol$^+$/trol$^+$* or *trol$^-$/trol$^-$* cell boundaries was performed on CpC or TF cells of LL3 gonads. *trol$^-$* cells localise significantly lower *D*E-cadherin amounts at their surfaces than *trol$^+$* cells. To allow for paired comparisons, measurements were taken from neighbouring control and experimental cells. P values of two-tailed, paired t-tests considered statistically significant between control and experimental samples are indicated (**: P≤0.005). The mean (cross) and median (line across box) for each of the samples are shown. We quantified 16 *trol$^+$* and 16 *trol$^-$* CpC or TF cell boundaries from 9 gonads. Clones were induced using the *hs-flp/FRT* system. **(E)** Percentage of control and experimental germaria showing displaced CpCs. **(F)** Quantification of the number of GSCs per niche and distribution of germaria containing 0-1 or 2-4 GSCs in control and experimental germaria. P values of two-tailed, unpaired t-tests considered statistically significant between control and experimental samples are indicated (*: P≤0.05, **: P≤0.005, ***: P≤0.0005). Numbers in bars refer to number of germaria analysed. Scale bars = 10µm.
